# Association of maternal prenatal selenium concentration and preterm birth: a multicountry meta-analysis

**DOI:** 10.1136/bmjgh-2021-005856

**Published:** 2021-09-09

**Authors:** Nagendra Monangi, Huan Xu, Rasheda Khanam, Waqasuddin Khan, Saikat Deb, Jesmin Pervin, Joan T Price, INTERBIO-21st Study Consortium, Stephen H Kennedy, Abdullah Al Mahmud, Yuemei Fan, Thanh Q Le, Angharad Care, Julio A Landero, Gerald F Combs, Elizabeth Belling, Joanne Chappell, Fansheng Kong, Criag Lacher, Salahuddin Ahmed, Nabidul Haque Chowdhury, Sayedur Rahman, Furqan Kabir, Imran Nisar, Aneeta Hotwani, Usma Mehmood, Ambreen Nizar, Javairia Khalid, Usha Dhingra, Arup Dutta, Said Ali, Fahad Aftab, Mohammed Hamad Juma, Monjur Rahman, Bellington Vwalika, Patrick Musonda, Tahmeed Ahmed, Md Munirul Islam, Ulla Ashorn, Kenneth Maleta, Mikko Hallman, Laura Goodfellow, Juhi K Gupta, Ana Alfirevic, Susan Murphy, Larry Rand, Kelli K Ryckman, Jeffrey C Murray, Rajiv Bahl, James A Litch, Courtney Baruch-Gravett, Zarko Alfirevic, Per Ashorn, Abdullah Baqui, Jane Hirst, Cathrine Hoyo, Fyezah Jehan, Laura L Jelliffe-Pawlowski, Anisur Rahman, Daniel E Roth, Sunil Sazawal, Jeffrey Stringer, Ge Zhang, Louis Muglia

**Affiliations:** 1 Division of Neonatology, Cincinnati Children's Hospital Medical Center, Cincinnati, Ohio, USA; 2 Center for Prevention of Preterm Birth, Perinatal Institute, Cincinnati Children’s Hospital Medical Center and March of Dimes Prematurity Research Center Ohio Collaborative, Cincinnati, Ohio, USA; 3 Department of Pediatrics, University of Cincinnati College of Medicine, Cincinnati, Ohio, USA; 4 Division of Human Genetics, Cincinnati Children's Hospital Medical Center, Cincinnati, Ohio, USA; 5 International Health, Johns Hopkins University Bloomberg School of Public Health, Baltimore, Maryland, USA; 6 Biorepository and Omics Research Group, Department of Pediatrics and Child Health, Faculty of Health Sciences, Medical College, The Aga Khan University, Karachi, Sindh, Pakistan; 7 Center for Public Health Kinetics, New Delhi, India; 8 Research Division, Public Health Laboratory, Center for Public Health Kinetics, Chake Chake, Tanzania; 9 Maternal and Child Health Division, International Centre for Diarrhoeal Disease Research Bangladesh, Dhaka, Dhaka District, Bangladesh; 10 Obstetrics and Gynecology, University of North Carolina at Chapel Hill, Chapel Hill, North Carolina, USA; 11 INTERBIO-21st Study Consortium, Nuffield Department of Women’s & Reproductive Health, University of Oxford, Oxford, UK; 12 Nutrition and Clinical Services Division, International Centre for Diarrhoeal Disease Research Bangladesh, Dhaka, Dhaka District, Bangladesh; 13 Center for Child Health Research, Faculty of Medicine and Health Technology, Tampere University, Tampere, Pirkanmaa, Finland; 14 Benh Vien Tu Du, Ho Chi Minh City, Viet Nam; 15 Department of Women's and Children's Health, University of Liverpool, Liverpool, UK; 16 Department of Chemistry, University of Cincinnati, Cincinnati, Ohio, USA; 17 Jean Mayer USDA Human Nutrition Research Center on Aging, Tufts University, Medford, Massachusetts, USA; 18 Grand Forks Human Nutrition Research Center, USDA ARS, Grand Forks, North Dakota, USA; 19 Projahnmo Research Foundation, Dhaka, Bangladesh; 20 Nutritional and Clinical Services Division, International Centre for Diarrhoeal Disease Research Bangladesh, Dhaka, Dhaka District, Bangladesh; 21 School of Medicine, University of Zambia, Lusaka, Zambia; 22 School of Public Health, University of Zambia, Lusaka, Zambia; 23 Cardiology, ICDDR, B, Dhaka, Bangladesh; 24 Nutrition and Clinical Services Division, International Centre for Diarrhoeal Disease Research Bangladesh, Dhaka, Bangladesh; 25 University of Tampere, Tampere, Pirkanmaa, Finland; 26 School of Public Health, University of Malawi College of Medicine, Blantyre, Malawi; 27 Medical Research Centre Oulu, PEDEGO Research Unit, University of Oulu, Oulu, Pohjois-Pohjanmaa, Finland; 28 Department of Women's and Children's Health, University of Liverpool, Liverpool, Merseyside, UK; 29 Department of Obstetrics and Gynecology, Duke University Medical Center, Durham, North Carolina, USA; 30 Department of Obstetrics, Gynecology and Reproductive Sciences, University of California San Francisco, San Francisco, California, USA; 31 Department of Epidemiology, University of Iowa, Iowa City, Iowa, USA; 32 Department of Pediatrics, University of Iowa, Iowa City, Iowa, USA; 33 Department of Medicine, World Health Organization, Geneva, Switzerland; 34 Global Alliance to Prevent Prematurity and Stillbirth, Lynnwood, Washington, USA; 35 Division of Perinatal Medicine, University of Liverpool, Liverpool, UK; 36 Center for Child Health Research, Faculty of Medicine and Health Technology, University of Tampere, Tampere, Pirkanmaa, Finland; 37 Department of Pediatrics, Tampere University Hospital, Tampere, Finland; 38 International Center for Maternal and Newborn Health, Department of International Health, Johns Hopkins University Bloomberg School of Public Health, Baltimore, Maryland, USA; 39 Nuffield Department of Women’s and Reproductive Health, University of Oxford, Oxford, UK; 40 Department of Biological Sciences and Center for Human Health and the Enivironment, North Carolina State University, Raleigh, North Carolina, USA; 41 Department of Pediatrics and Child Health, Aga Khan University, Karachi, Pakistan; 42 Department of Epidemiology and Biostatistics, University of California San Francisco, San Francisco, California, USA; 43 Department of Paediatrics, The Hospital for Sick Children, Toronto, Ontario, Canada; 44 Burroughs Wellcome Fund, Research Triangle Park, North Carolina, USA

**Keywords:** child health, environmental health, epidemiology, maternal health, nutrition

## Abstract

**Background:**

Selenium (Se), an essential trace mineral, has been implicated in preterm birth (PTB). We aimed to determine the association of maternal Se concentrations during pregnancy with PTB risk and gestational duration in a large number of samples collected from diverse populations.

**Methods:**

Gestational duration data and maternal plasma or serum samples of 9946 singleton live births were obtained from 17 geographically diverse study cohorts. Maternal Se concentrations were determined by inductively coupled plasma mass spectrometry analysis. The associations between maternal Se with PTB and gestational duration were analysed using logistic and linear regressions. The results were then combined using fixed-effect and random-effect meta-analysis.

**Findings:**

In all study samples, the Se concentrations followed a normal distribution with a mean of 93.8 ng/mL (SD: 28.5 ng/mL) but varied substantially across different sites. The fixed-effect meta-analysis across the 17 cohorts showed that Se was significantly associated with PTB and gestational duration with effect size estimates of an OR=0.95 (95% CI: 0.9 to 1.00) for PTB and 0.66 days (95% CI: 0.38 to 0.94) longer gestation per 15 ng/mL increase in Se concentration. However, there was a substantial heterogeneity among study cohorts and the random-effect meta-analysis did not achieve statistical significance. The largest effect sizes were observed in UK (Liverpool) cohort, and most significant associations were observed in samples from Malawi.

**Interpretation:**

While our study observed statistically significant associations between maternal Se concentration and PTB at some sites, this did not generalise across the entire cohort. Whether population-specific factors explain the heterogeneity of our findings warrants further investigation. Further evidence is needed to understand the biologic pathways, clinical efficacy and safety, before changes to antenatal nutritional recommendations for Se supplementation are considered.

**Key questions**

**What is already known?**

- Conflicting results have been reported on the association between maternal selenium (Se) levels and preterm birth (PTB) risk.
- Most previous studies were typically small or focused on a single geographic region with limited data in populations at high risk for PTB.

**What are the new findings?**

- Maternal prenatal plasma/serum Se concentrations varied substantially between different geographic regions.Clinically meaningful associations were observed between maternal Se concentration and PTB within specific cohorts; however, this finding was not generalisable across all the cohorts.
- The significant associations observed in specific study cohorts might be mediated or confounded by individual site-specific factors.

**What do the new findings imply?**

- Our results do not support a uniform association between maternal prenatal Se concentration and PTB risk.
- The significant associations observed in specific study cohorts might have potential implications for targeted Se supplementation in hig-risk settings.

## Introduction

Preterm birth (PTB), defined as delivery prior to 37 completed weeks of gestation, is the leading global cause of infant and under-5-year old childhood mortality.^1^ Each year, an estimated 15 million babies are born preterm, of whom approximately 1 million die with complications of prematurity.^2^ Most countries with reliable trend data show an increase in PTB rates over the past 20 years, with more than 60% of cases occurring in Africa and South Asia.^2–4^ Due to the immaturity of multiple organ systems, preterm infants are at increased risk of short-term and long-term health sequelae including cognitive disabilities, impaired motor skills, hearing loss, chronic immunologic/infectious morbidities^5 6^ and elevated risks of adulthood obesity, diabetes and hypertension.^7–9^

Despite the profound global health significance and recognition that the prevention of PTB would provide major improvements in child health, there has only been limited progress in preventing PTB. Recently, a two-stage genome-wide association study of over 50 000 women of European ancestry identified and replicated EEFSEC gene, encoding the selenocysteine tRNA (tRNA^Seleno^)-specific eukaryotic elongation factor, that was robustly associated with gestational duration.^10^ EEFSEC plays a critical role in incorporating selenium (Se) in the form of selenocysteine into selenoproteins such as glutathione peroxidases, the iodothyronine 5′-deiodonases, selenoprotein P and thioredoxin reductases.^11^ The implication of selenocysteine pathway suggests a potential benefit for further evaluation of the role of maternal Se status on PTB risk. The possible involvement of maternal Se concentration in PTB has also been suggested by previous epidemiological studies;^12–16^ however, the sample sizes of these studies were usually small, or focus on a single geographic area and the results are not always consistent between studies.^17 18^

In this study, we aimed to examine the association of maternal Se concentrations during pregnancy with PTB risk and gestational duration. As dietary Se intake is highly related to its regional soil content,^19^ we leveraged the availability of archived biological samples from geographically diverse cohorts and tested the association between maternal Se concentrations and gestational duration in a large number of samples collected from these study cohorts with different social and ancestral background and varying degrees of Se exposures.

## Methods

### Study design and participants

**Figure 1:**
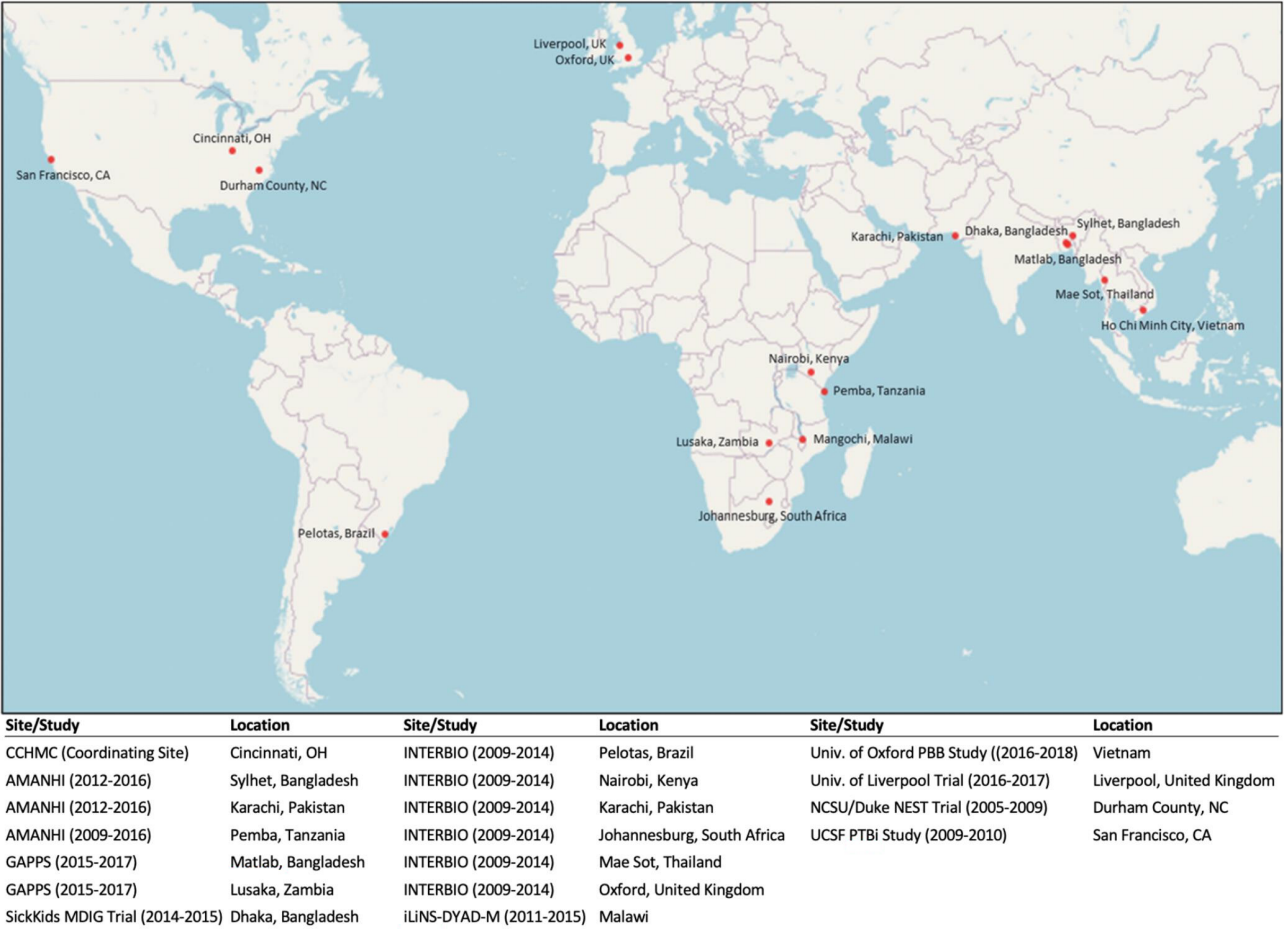
Geographic location of study sites.

The International Consortium on Selenium, Genetics, and Preterm Birth is a Bill & Melinda Gates Foundation (BMGF) funded project to study the potential association between maternal Se concentration and PTB risk using existing samples and data from multiple birth studies. The consortium comprises 17 international pregnancy cohorts across a wide geographic distribution (figure 1) with Cincinnati Children’s Hospital Medical Center (CCHMC) serving as the coordinating hub. Among the participating sites, Malawi (iLiNS-DYAD)^20^ and Bangladesh (MDIG)^21^ cohorts were intervention trials and USA, CA (CPPOP) was a case–control study. All the other cohorts were designed to enrol women randomly at hospitals. Description and study characteristics of these participating study cohorts are provided in online supplemental text 1 and online supplemental table 1).

10.1136/bmjgh-2021-005856.supp1 Supplementary data

### Samples and sampling data

Demographic, prenatal, delivery and fetal/newborn data (online supplemental table 2) as collected by the individual sites according to their local protocols were shared with the coordinating hub (CCHMC). The data collected from Bangladesh (GAPPS), Bangladesh (MDIG),^21^ Vietnam (PBB), USA (NEST; CPPOP),^22 23^ and all AMANHI cohorts^24^ were case/control (preterm/term) samples. The data collected from other sites including Malawi (iLiNS-DYAD),^20^ Zambia (GAPPS) and the six INTERBIO sites^25^ were random samples with a PTB rate ranging from 4.5% (INTERBIO, Kenya) to 20% (INTERBIO, Pakistan). Gestational age dating was assigned at the site level by ultrasound, last menstrual period (LMP) or both (online supplemental table 1). Preterm cases were defined as birth prior to 37 weeks of gestation and term controls as birth at 37 weeks or later. We excluded stillbirths and multigestational pregnancies.

### Selenium measurement

Se status was assessed on the basis of the concentrations of Se in plasma or serum.^26^ Plasma or serum samples obtained from participating cohorts were stored at −70°C or −80°C refrigerators before and after use at the CCHMC Biobank (online supplemental table 1). To mitigate potential batch effect, samples from each site were randomised prior to analysis in batches. Inductively coupled plasma mass spectrometry (ICP-MS) measurements of Se concentrations in serum or plasma were performed using Agilent 7700 ICP-MS (Agilent Technologies) at the laboratory of Clinical Chemistry and Biochemistry, University of Cincinnati as described in detail in the protocol (online supplemental text 2) except the samples from Bangladesh (MDIG) which were analysed at the Centers for Disease Control and Prevention (Atlanta, GA).

### Statistical analysis

Phenotypic data from participating study sites were harmonised by applying a uniform data structure and consistent coding rules for phenotype variables (eg, gestational duration, maternal age, height and fetal sex). Maternal Se data generated by the laboratory were combined and merged with phenotype data. The distributions of gestational duration and Se measures for each site were visually inspected using histograms and violin plots. Outliers for gestational duration and Se measurements were detected based on fitting with appropriate probability distributions and removed from further association analysis.

To determine the covariates to be included in the association analysis, we first examined the correlation of PTB (and gestational duration) with other covariates as well as the correlation between Se concentration and other covariates in each site using Pearson correlation. Variables significantly correlated (p<0·05) with either PTB or gestational duration or Se concentration were included as covariates. The DerSimonian-Laird (DSL) random-effect meta-analysis was used to combine the correlation coefficients obtained from each cohort. For each site, we estimated the association between maternal Se concentration and PTB (and gestational duration as a continuous variable) using logistic (for PTB) or linear (for gestational duration) regression analysis. Fixed-effect meta-analysis and random-effect meta-analysis were used to combine the results from different cohorts. Between-study heterogeneity was checked using Cochran’s Q test. Some of the cohorts used case/control samples (online supplemental table 1) and regression analysis of gestational duration as a continuous variable without accounting for the non-random sampling could potentially introduce bias in effect size estimation. To address this problem, we conducted regression analysis weighted by inverse of sampling probability (IPW). Detailed description of this analysis can be found in online supplemental text. All analyses were done with Microsoft R Open 3.5.1.

### Patient and public involvement

Patients or public were not involved in setting the research question or the outcome measures, nor were they involved in the design or conduct of the study. No participants were asked to advise on interpretation or writing up of the manuscript. For the study, individual study cohorts shared the archived biological samples from established biobanks and there is no direct patient or public involvement.

## Results

### Gestational duration, PTB and their correlations with other covariates

**Figure 2:**
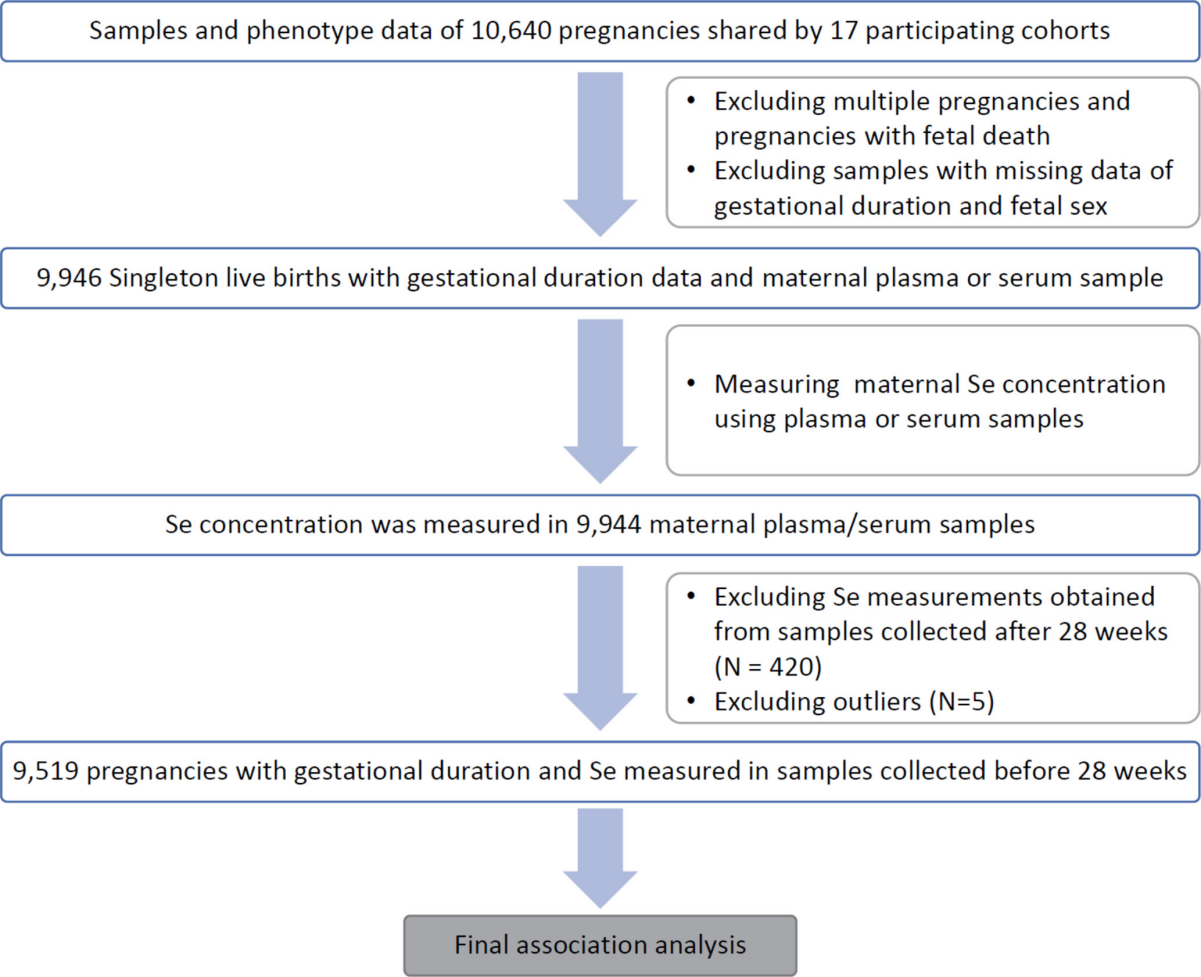
Flow chart of the study illustrating the total number of subjects, inclusion/exclusion criteria.

Pregnancy phenotype and birth outcomes of 10 640 pregnancies were obtained from 17 study sites (online supplemental table 1). Among these, 9946 singleton livebirths had gestational duration measured in days (gday) and maternal plasma or serum samples (figure 2). The demographic characteristics of these mothers (eg, age and height) and the major birth outcomes (eg, gestational duration and birth weight) are summarised by the site (table 1). After removing three outliers, the gestational duration followed a Weibull distribution with a mean of 268 days and ranging from 147 to 312 days (distribution parameters: shape: 21.2, scale: 275.8) (online supplemental figure 1). The distributions of gestational days in term (gday ≥259 days) and preterm (gday <259 days) deliveries from each site are shown in online supplemental figure 2.

**Table 1:** Demographic characteristics of study subjects

Site	Sample size	Term	Preterm	Male	Female	Gday at delivery	Gday at sampling	Maternal age (year)	Maternal height (cm)	Birth weight (g)
Bangladesh (AMANHI)	506	253 (50%)	253 (50%)	239 (47%)	267 (53%)	260.7 (20)	95.8 (23.1)	23.6 (4.5)	149.2 (5.6)	2516.1 (495.2)
Bangladesh (GAPPS)	258	172 (67%)	86 (33%)	132 (51%)	126 (49%)	267.8 (18.7)	158.8 (4.7)	23.9 (5.9)	151.8 (5.5)	2722.7 (581.1)
Bangladesh (MDIG)	208	138 (66%)	70 (34%)	106 (51%)	102 (49%)	265.9 (14.2)	143.9 (13.5)	23.4 (4.5)	151.4 (5.6)	2664.2 (375.7)
Brazil (INTERBIO)	389	344 (88%)	45 (12%)	212 (54%)	177 (46%)	270.4 (10.8)	132.2 (53.9)	28.5 (5.4)	162.5 (6.4)	3147.1 (464.3)
Kenya (INTERBIO)	553	528 (96%)	25 (4%)	293 (53%)	260 (47%)	278.3 (11.1)	112.5 (40.6)	30.4 (4.1)	161.8 (5.8)	3267 (463.8)
Malawi (iLiNS-DYAD)	1212	1126 (93%)	86 (7%)	587 (48%)	625 (52%)	276 (14.3)	117.7 (14.9)	25.2 (6.2)	156.1 (5.7)	2976.6 (449.5)
Pakistan (AMANHI)	348	233 (67%)	115 (33%)	189 (54%)	159 (46%)	265.5 (16.5)	95.1 (24.6)	26.3 (5.1)	154.8 (6.1)	2684.4 (500.1)
Pakistan (INTERBIO)	516	413 (80%)	103 (20%)	251 (49%)	265 (51%)	264.9 (13.5)	103.5 (35.6)	30.1 (4.6)	158 (5.9)	2876.5 (480.7)
South Africa (INTERBIO)	352	299 (85%)	53 (15%)	181 (51%)	171 (49%)	269.4 (17.5)	88.2 (19.6)	30.2 (5.8)	159 (6.9)	2940.3 (588.8)
Tanzania (AMANHI)	351	234 (67%)	117 (33%)	174 (50%)	177 (50%)	267.5 (19.6)	99.1 (23.1)	27.9 (6.6)	155.2 (5.9)	3111.9 (592.5)
Thailand (INTERBIO)	514	485 (94%)	29 (6%)	266 (52%)	248 (48%)	275.6 (11.5)	114.4 (37.1)	26.2 (6.1)	151.8 (5.1)	2965.8 (457.3)
UK (INTERBIO)	648	594 (92%)	54 (8%)	342 (53%)	306 (47%)	275.9 (14.6)	89.9 (20.3)	31.1 (4.8)	165.3 (6.5)	3301.1 (586)
UK (Liverpool)	525	424 (81%)	101 (19%)	271 (52%)	254 (48%)	267 (21.7)	140.8 (9.5)	30.6 (4.9)	164.8 (6.3)	3141.2 (730.3)
USA, California (CPPOP)	966	484 (50%)	482 (50%)	505 (52%)	461 (48%)	249.8 (29.9)	115.7 (7.7)	30 (6.1)	161.6 (7.3)	2763.1 (923)
USA, North Carolina (NEST)	657	438 (67%)	219 (33%)	363 (55%)	294 (45%)	263.1 (22.5)	161.1 (90.5)	28.3 (6.1)	162.8 (7.7)	2999.6 (750.4)
Vietnam (PBB)	970	651 (67%)	319 (33%)	495 (51%)	475 (49%)	264.4 (18.9)	149 (6.2)	29.1 (4.6)	156 (4.8)	2959.9 (613.5)
Zambia (GAPPS)	973	853 (88%)	120 (12%)	478 (49%)	495 (51%)	271.6 (18.2)	137.3 (27.2)	27.7 (5.8)	160.4 (6.5)	3008.7 (591.7)
All	9946	7669 (77.1%)	2277 (22.9%)	5084 (51.1%)	4862 (48.9%)	267.8 (20.2)	122.1 (39.9)	28 (5.9)	158.5 (7.6)	2967.4 (637.4)

We examined the correlation of PTB and gestational duration with other covariates (maternal age, height, fetal sex and gestational age at sampling) in each participant site (online supplemental figure 3). Meta-analysis using DSL method showed that PTB risk was significantly associated with maternal height and fetal sex. Similarly, gestational duration was also significantly associated with maternal height (shorter mothers had shorter gestational duration) and fetal sex (males had shorter gestational duration).

### Maternal prenatal Se concentration and its correlations with other covariates

**Figure 3:**
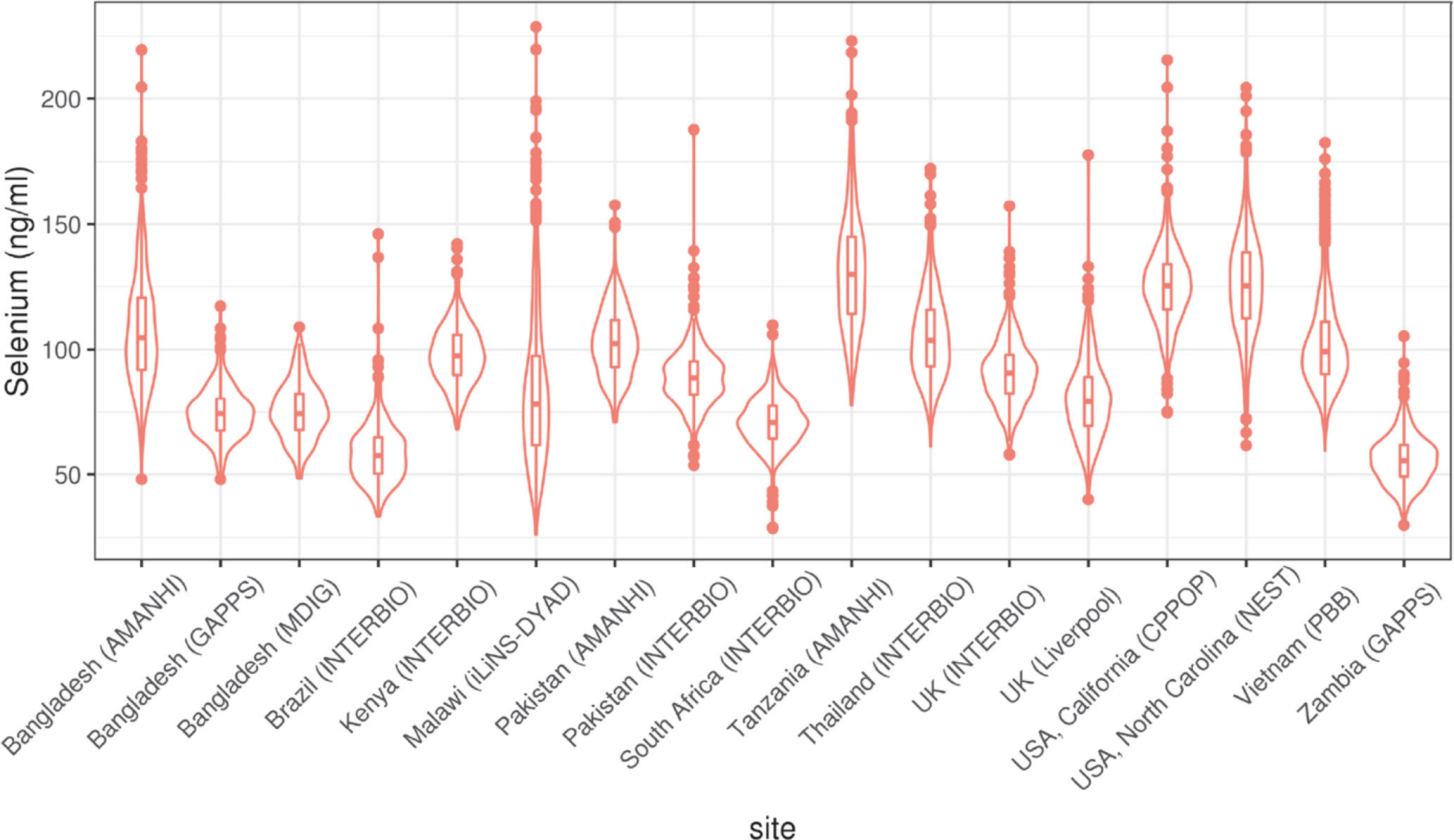
Selenium concentration by participating sites.

Se concentrations were successfully measured in 9944 mothers. After removing two outliers, the Se concentrations followed a normal distribution with some positive skewness (online supplemental figure 4) with a mean of 93.8 ng/mL and SD of 28.5 ng/mL. Se levels varied substantially across different sites (figure 3, online supplemental figure 5), and also across different experimental batches for each site (online supplemental figure 6). The highest average Se was observed in the Tanzania (AMANHI) cohort with a mean level of 131.4 ng/mL, and the lowest Se was observed in Zambia (GAPPS)with a mean concentration of 55.9 ng/mL. The largest variation was observed in Malawi (iLiNS-DYAD) (range: 26.1 to 228.7 ng/mL, SD=29.5) (online supplemental table 3).

We examined the correlation of maternal Se concentration with other covariates in each site. When combined across sites, the Se concentration across sites was significantly positively correlated with maternal age (ρ=0·08, p=2.5e−5) and negatively correlated with gestational age at the time of sample collection (ρ = −0.13, p=3.0e−5) (online supplemental figure 7). The gestational age at sample collection varied substantially from site to site and in some sites, there were some samples collected after second trimester (≥28 weeks). In order to minimise the bias introduced by these samples (eg, exclusion of extremely PTB and reduction of maternal Se concentration), we excluded 416 samples which were collected at 28 weeks of gestational age or later and four samples without known date of sample collection) from the final association analysis (online supplemental figure 8).

### Association of maternal selenium concentration with PTB and gestational duration

**Figure 4:**
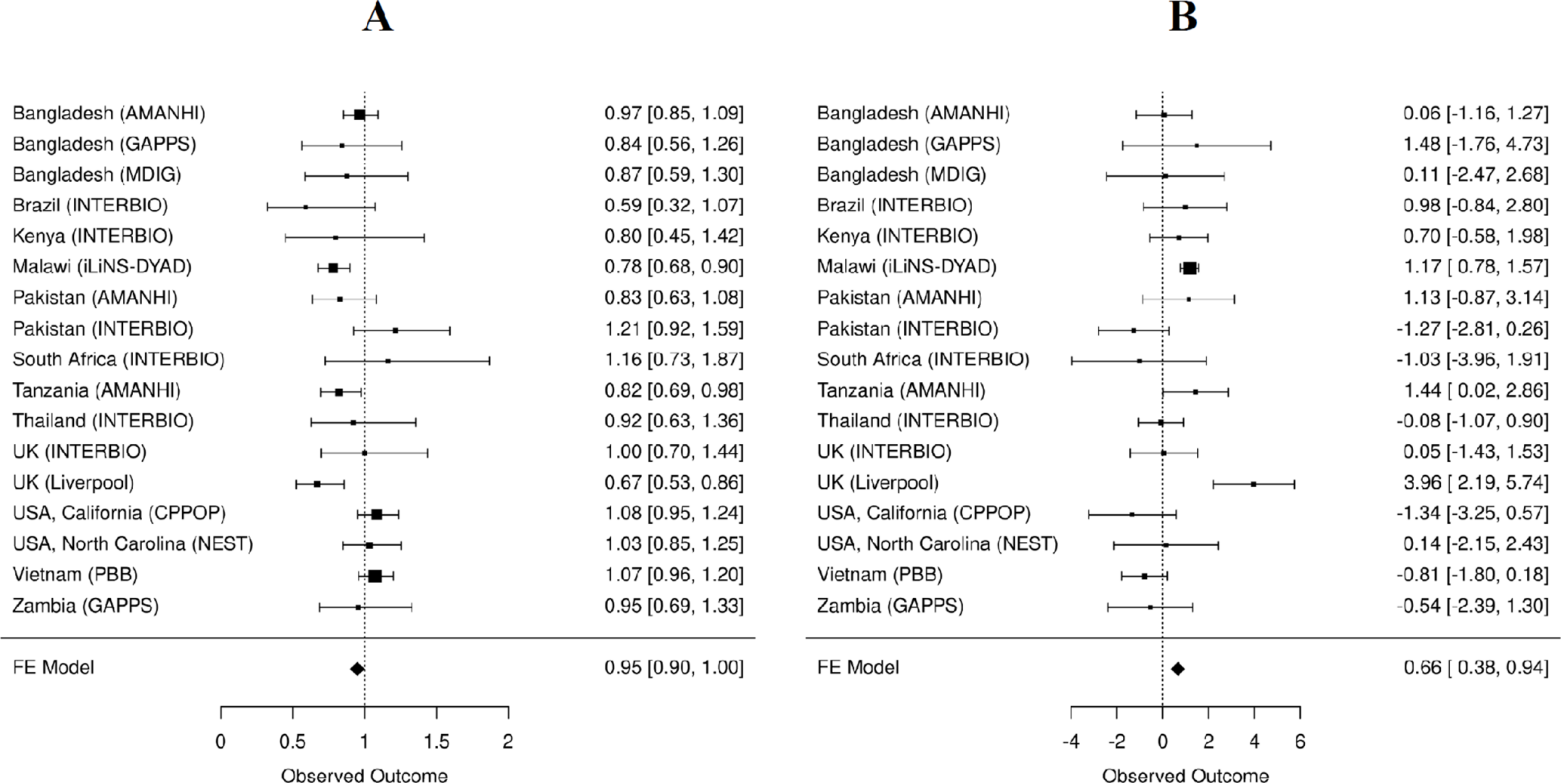
Meta-analysis of the association of maternal Se concentration with PTB (A) and gestational duration (B). (A) The estimated association between Se concentration and PTB is shown as OR per 15 ng/mL increase in Se concentration. (B) The estimated association between Se concentration and gestational duration is shown as change in gestational days per 15 ng/mL increase in Se concentration. PTB, preterm birth; Se, selenium.

We examined the association of maternal prenatal Se (before third trimester with gestational duration at sample collection <28 weeks) with PTB and gestational duration in each individual site and then combined the results using meta-analysis (figure 4). In total, the associations were tested in 9519 pregnancies (figure 2). The following factors found to be significantly associated (p<0.05) with either gestational duration or Se concentration were incorporated as covariates. These include maternal age (mage), maternal height (ht), fetal sex (fsex) and gestational days at sample collection (gday(sample)) and experimental batch (batch).

The fixed-effect meta-analysis across the 17 cohorts showed that Se concentration was significantly associated with PTB and gestational duration. The associated effect size estimates were an OR=0.95 (95% CI: 0.9 to 1.00) for PTB or 0.66 days (95% CI: 0.38 to 0.94) longer gestation per 15 ng/mL increase in Se concentration. However, there was substantial between cohort heterogeneity as shown by the forest plots (figure 4) and the significant p values for Cochran’s Q statistic (p=0.0037 for PTB and p=6.03e−5 for gestational duration). Given the enrichment of preterm cases in the case–control studies that could potentially introduce bias, we conducted the IPW analysis in the eight case/control data sets and the results were similar to the meta-analysis of gestational age without adjustments (online supplemental figure 9).

The largest effect sizes were observed in UK (Liverpool) cohort, and highly significant associations were observed in Malawi (iLiNS-DYAD) samples (figure 4). Other than these two, only the Tanzania (AMANHI) cohort showed associations with marginally smaller p-values (PTB: p=0.026 and gestational duration: p=0.049). After excluding the Malawi (iLiNS-DYAD) and UK (Liverpool) cohorts, the fixed-effect meta-analysis was no longer significant (PTB: p=0.82 and gestational duration: p=0.92). Given the large between study heterogeneity, we also conducted a random-effect meta-analysis of all 17 cohorts. The associations of maternal Se concentrations with PTB and gestational duration in this model did not achieve statistical significance (PTB: p=0.081 and gestational duration: p=0.24).

### Stratified analysis of Malawi cohort

**Figure 5:**
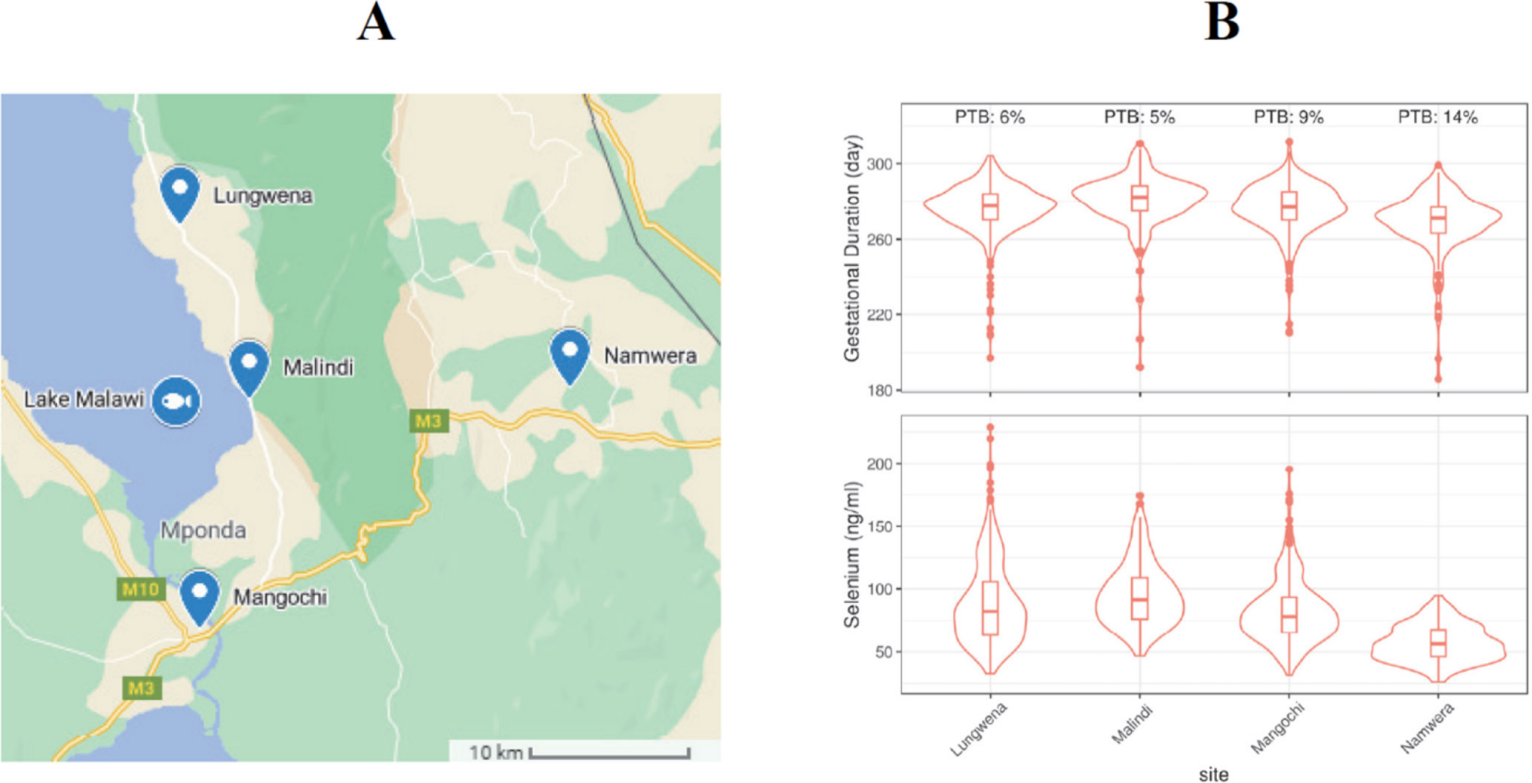
Geographic distribution of the four Malawi subsites (A) and distributions of gestational duration and maternal Se concentration at these four sample recruitment sites (B). PTB, preterm birth; Se, selenium.

As noted above, the Malawi (iLiNS-DYAD) cohort showed the most significant associations between Se concentrations and PTB (p=0.00062) and gestational duration (p=7.7e−9) (figure 4). Given these findings, we attempted to investigate possible factors that drove these associations. Participants of the Malawi cohort were enrolled from four health facilities that covered mostly one continuous area near Lake Malawi (figure 5A). Lungwena, Malindi and Mangochi subsites are along the banks of Lake Malawi and close to Namizimu forest reserve. The Namwera subsite is in the mountains and relatively distant from the other three sites. The demographic characteristics of the mothers (eg, age and height) and the major birth outcomes (eg, gestational duration and birth weight) separated by geographic distribution were summarised by the subsites (online supplemental table 4). Compared with the other sites, participants from Namwera had a higher PTB rate (14%) and lower mean gestational duration and birth weight (figure 5B and online supplemental table 4). The lowest mean Se (mean=56.8 ng/mL and SD=14.4 ng/mL) was also observed in Namwera samples (figure 5B and online supplemental table 5). When subsite of sample collection was included as a covariate the effect size estimations of maternal Se concentration were OR=0.85 (95% CI: 0.72 to 1.00) for PTB and 0.49 longer days of gestation (95% CI: 0.07 to 0.92) per 15 ng/mL increase in Se concentration (online supplemental figure 10). Although still significant, these estimates were smaller than the estimates obtained without adjustment for subsites (OR=0.78 (CI: 0.68 to 0.90) or 1.17 days (95% CI: 0.78 to 1.57)) (figure 4).

### Stratified analysis of UK (Liverpool) cohort

**Figure 6:**
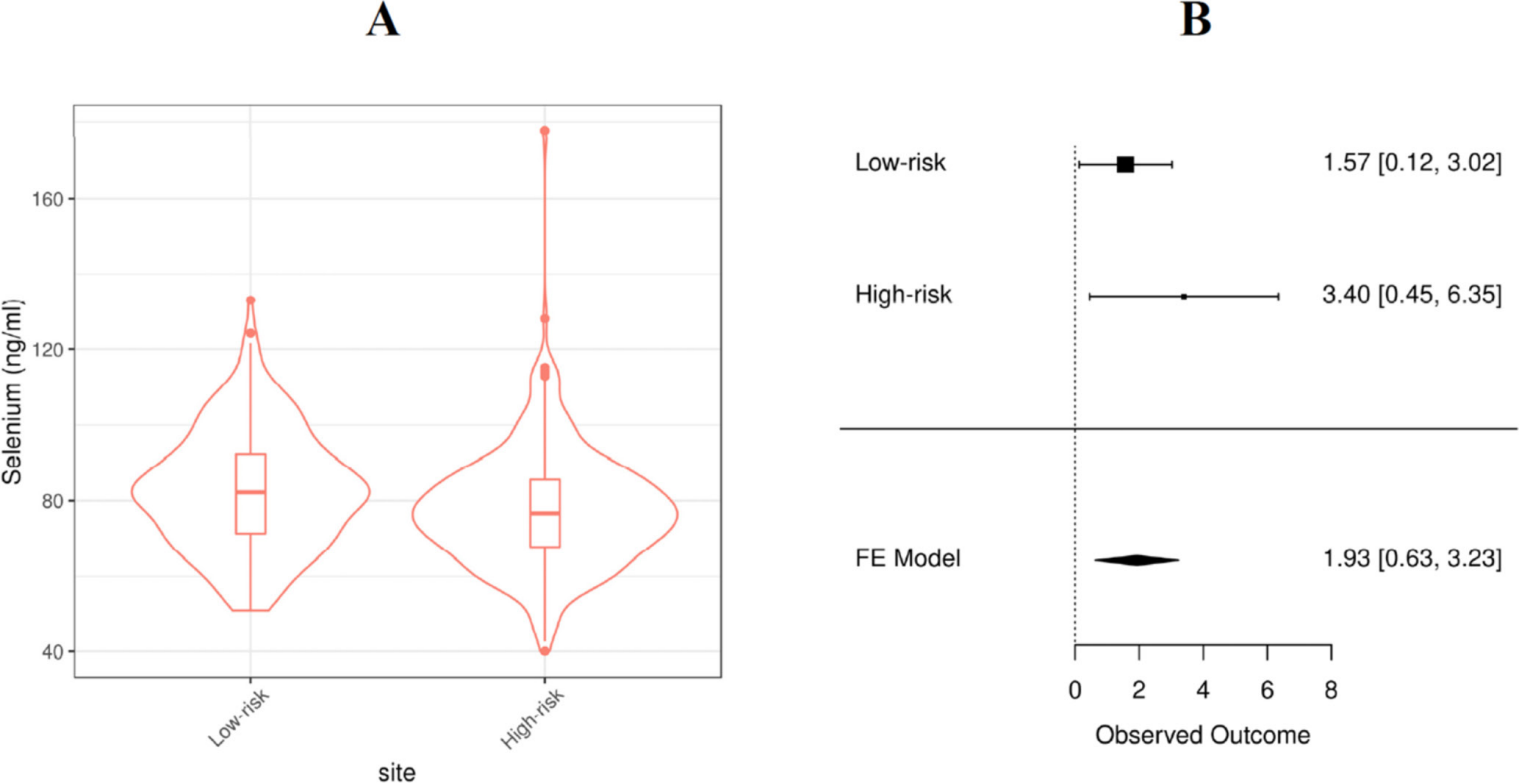
Selenium concentrations of Liverpool cohort (A) and the meta-analysis of maternal Se concentration associated with gestational duration (B). Se, selenium.

The largest effect sizes for Se concentration on gestational duration (3.96 days longer gestation per 15 ng/mL increase in Se) or PTB risk (OR=0.67 per 15 ng/mL increase in Se) were observed in the Liverpool cohort (figure 4). This cohort included 272 high-risk mothers who had a previous PTB and 253 low-risk mothers who did not have previous history of PTB. Among the 272 high-risk mothers, 97 (36%) of them had a subsequent PTB (online supplemental table 6). The average Se concentration was lower in the high-risk mothers than the low-risk mothers (mean=77.6 vs 82.7, t-test p=0.0002) (figure 6A and online supplemental table 7). In both low-risk and high-risk groups, Se concentration was positively associated with gestational duration (1.93 days (95% CI: 0.63 to 3.23) per 15 ng/mL increase in Se concentration (figure 6B)) and Se concentration was also associated with PTB risk in the high-risk group (OR=0.74 per 15 ng/mL increase in Se concentration, CI: 0.56 to 0.98).

## Discussion

In this multicountry study conducted in low-resource Asian and African countries with high rates of PTB risk and high-resource European and US contexts, we studied the maternal prenatal Se concentrations and their association with the risk of PTB and gestational duration. We found that the Se concentrations varied substantially across different study cohorts. The highest levels of maternal Se were observed in samples collected from Pemba Island (Tanzania) and in the USA (California (CPPOP) and North Carolina (NEST) cohorts. The lowest levels of maternal Se were observed in Zambia (GAPPS) and Brazil (INTERBIO). The average levels of maternal Se in the two cohorts with the lowest levels of maternal Se (<60 ng/mL) were less than half of the average levels observed in the cohorts with the highest levels of maternal Se (>125 ng/mL) (figure 3).

### Possible explanation of our findings

Our result demonstrates the substantial variation in Se between different geographic regions. However, even among sites in geographic and cultural proximity (eg, the three Bangladesh or the two Pakistan sites), the maternal Se levels were still different, indicating factors other than geographic location likely influenced maternal Se levels. We also found that the Se concentration positively correlated with maternal age and negatively correlated with gestational age at the time of sample collection. It is unknown whether the decrease of maternal Se reflects the overall increased consumption of nutrients during pregnancy or if Se is utilised by specific biological processes in pregnancy. Also, it is unclear if the decrease in maternal Se with gestational age is due to relative haemodilution because of progressively greater expansion of plasma volume than the increase in red blood cell mass.

In the association analysis between maternal Se concentrations (before third trimester), PTB and gestational duration, we obtained heterogeneous results from the 17 cohorts (figure 4). We observed significant negative associations between maternal Se and PTB risk and positive associations with gestational duration in Malawi (iLiNS-DYAD), UK (Liverpool) and Tanzania (AMANHI) cohorts. However, the associations were not significant in other cohorts and the random-effect meta-analysis of the 17 cohorts altogether also did not show significant associations.

Further stratified analysis of the Malawi (iLiNS-DYAD) cohort based on the geographic locations of the four sample collection sites (within 30 km apart, figure 5A) suggested that the observed association between Se concentration with PTB and gestational duration was mainly driven by the Namwera site (figure 5B). This site had participants with the highest PTB rate and shortest gestational duration, and lowest Se concentration compared with other three sites near the coast of Lake Malawi. After adjustment for the site of sample collection, the estimated effect size and significance of the associations substantially attenuated. This result suggests that there may be some site-specific confounding factors. However, it is also possible that the low Se concentration is a driving factor that causes the high PTB rate and shorter gestational duration in the Namwera samples because in Namwera samples alone, Se concentration is significantly associated with PTB risk and gestational duration.

The association with the largest effect size between Se and gestational duration was observed in the UK (Liverpool) cohort which included a high-risk and a low-risk group of mothers based on their previous history of PTB. The Se concentration was significantly different between these two groups (figure 6A), which suggests the Se concentration might be an indicator of some long-term risk for PTB which may have also had an effect on previous pregnancies in this cohort. Within each of the UK (Liverpool) groups, maternal Se concentration was associated with the gestational duration of the current pregnancies (figure 6B). In another UK cohort (UK INTERBIO collected at Oxford), the mean Se concentration was approximately 10 ng/mL higher but was not associated with PTB risk or gestational duration. These disparate findings even between two UK cohorts suggests that unmeasured site-specific factors are either confounding or modifying the associations between maternal Se concentration and gestational duration.

### Comparison with other studies

Our findings contribute to an emerging literature focused on the association of Se status and pregnancy outcomes, especially the risk for PTB or gestational duration. Evidence supporting the potential involvement of Se in PTB risk includes a study of Dutch women in which the lowest quartile of serum Se had twice the risk of PTB as women in the upper three quartiles.^12^ Another study of pregnant women with HIV in Lagos, Nigeria showed significant associations observed between maternal Se deficiency and PTB.^13^ The Norwegian Mother, Father and Child Cohort study showed that higher Se intake from food was associated with increase in gestational length and decreased PTB risk.^15^ Furthermore, the Maternal Health and Birth outcomes study in South East Queensland, Australia, suggested that dietary Se concentrations were significantly higher in women birthing beyond 41 completed weeks of gestation in that cohort.^14^ However, there are also reports on Se metabolism with regard to gestational length that find contradicting results. The Japan Environment and Children’s Study (JECS) did not find an association between serum Se concentration and PTB risk.^17^ The Screening for Pregnancy Endpoints (SCOPE) study in Adelaide suggested that lower circulating levels of Se may be associated with a reduced risk of pregnancy complications including PTB risk.^16^ Of note is the fact that these previous studies mostly focus on a single geographic region, have limited or no Se deficiency and are generally based on small sample sizes.

Our study is the most extensive investigation of the association between mid-pregnancy Se concentration and the gestational duration and PTB in global populations, including several lower-income Asian and African countries with a very high baseline PTB risk. The diversity of our study participants and the wide distribution of study sites across different geographic regions enable us to draw some general conclusions. Overall, our results do not support a ubiquitous and strong association between maternal Se concentration and PTB risk. The lack of significant associations in the cohorts with low average Se concentration also suggests Se deficiency is not the primary factor influencing PTB risk. The significant associations observed in some study cohorts might be confounded or mediated by site-specific factors. For example, Se concentration might be associated with certain dietary patterns or socioeconomic status that drive the PTB risk or certain local factors that might interact with Se and jointly influence PTB risk in some high-risk pregnancies.

### Clinical importance

Several biologic mechanisms have been hypothesised to link Se status and PTB risk. Selenoproteins serve critical cellular homeostatic functions in maintaining redox status and antioxidant defenses, and modulate inflammatory responses, which have been linked to PTB.^27^ In some instances, preterm parturition is thought to be prompted by a cascade of inflammatory events, leading to cytokine upregulation and subsequent induction of uterine activity by promoting the expression and release of uterotonic factors.^28^ The essential micronutrient Se, which exerts its antioxidant and anti-inflammatory properties in the form of selenoproteins such as glutathione peroxidase 3, selenoprotein P1 and thioredoxin reductase, has been shown to be protective in various inflammatory-based disease models.^29–31^ A recent study in mice showed that Se in the form of selenoproteins played an indispensable role in uterine smooth muscle contractions, and the absence of any of these proteins affected the uterine contractility.^32^ In vitro study of Se supplementation demonstrated that selenite suppresses key mediators involved in inflammation-induced activation of mediators involved in active labour in human fetal membranes and the myometrium.^33^ Further investigations may benefit from looking in more detail at whether pregnancies exhibiting higher levels of inflammation or increased cytokine dysregulation benefit from higher levels of Se in terms of increased gestational duration or decreased PTB risk.

The hierarchy of biological activities of Se calls for biomarkers informative at different levels of Se exposure assessing Se intake, tissue Se, Se excretion and Se function.^26^ Plasma or serum Se level provides valuable information about the Se status over a wide range of Se intake; however, there is need for additional Se speciation information particularly for assessing Se status in non-deficient individuals for whom there is high risk for PTB. Epidemiological reports and research examining the effects of different Se species and their bioavailability and bioactivity especially during pregnancy are lacking. There are recent reports suggesting that the non-linear associations between whole blood Se and plasma Se may be primarily due to accumulation of large proportion of selenoneine in red blood cells especially in coastal populations consuming marine foods.^34^

### Strengths and limitations

There are some significant limitations of the current study. Of note is the fact that the samples and phenotypic data were retrieved from existing biorepositories collected several years ago in different studies. Although we harmonised and analysed a set of key variables known to be associated with PTB and gestational duration, we were not able to include some important environmental or socioeconomic factors in the analysis due to missing or incomplete data. We excluded stillbirth due to missing data on cause-of-death, under-reporting and lack of comparability in reporting of stillbirths, especially in low-income and middle-income countries regarding the birth weight and gestational age criteria. It will be key to include these variables across cohorts in future studies.

There were differences in how gestational age was determined and distributed across cohorts. Some cohorts determined the duration by ultrasound whereas others used LMP (or both). This different dating methodology between studies may have introduced some noise into the analysis. PTB rates reported in some low-income and middle-income cohort studies appear to be low, and this might be due to under-reporting and geographic location of the recruitment site. Also, some cohorts were enriched for PTB samples, and the distribution of gestational duration did not follow a normal distribution. Although regression analysis is generally robust regardless of meeting the normality assumption, this difference may have introduced some bias in these analyses. Also, we tested PTB as primary outcome using logistic regression, which is valid to both case–control and random samples. These issues certainly point to the importance of standardising dating and sampling methods as investigations move forward.

Also, of note with respect to the study limitations is that there was large variation in gestational age at when the plasma/serum samples were collected. Also, the samples were stored for different periods of time. Given the gestational age at sample collection significantly correlated with the Se concentration, we accounted for this variance by including only the pregnant mothers with samples collected before or during the second trimester and included gestational age at sample collection as a covariate in the final association analysis. Despite these methodological adaptations, it is possible that we may not have completely accounted for the influence of gestational age at sample collection if the effect is not completely linear. More standardisation with respect to timing of collection and storage times may simplify these types of analyses in future studies.

In addition, given that the major source of Se is food, and the large proportion of it comes from the staple food items such as rice, wheat and seafood,^19^ it is clear that studies looking at Se, PTB risk and gestational duration would benefit from more data on diet and nutrition. While information regarding the dietary intake of Se or other supplements during the pregnancy was not readily available for the majority of the enrolled subjects from the study sites, collection of such data in future studies would be hugely beneficial.

Finally, the generalisability of our results from the Se measurements to all study populations was likely limited due to the considerable variation in the local factors that influence the Se levels or modify the effect of Se during pregnancy. This highlights the need for larger coordinated studies examining extraneous factors that may be associated with Se levels, risk of PTB and gestational duration in pregnant women across different geographic settings.

## Conclusions

We studied maternal prenatal Se concentration and tested whether it is associated with the risk of PTB and gestational duration using data and samples collected from 17 international birth cohorts with diverse ethnic background and geographic distribution. Our study observed statistically significant associations between maternal Se concentration and PTB at some sites; however, this did not generalise across the entire cohort, which might lower the enthusiasm for wide use of Se supplements as a general strategy to prevent PTB or increase gestational duration. The significant associations observed in some cohorts and not others suggest local confounding factors or other risk modifiers. Effects of Se supplementation on PTB in high-risk populations with low Se in food (like Namwera region in Malawi) or in high-risk mothers with previous history of PTB need to be confirmed, ideally through a double-blind, placebo controlled clinical trial. Future studies that expand and refine sampling in populations that are found to have the greatest variations in Se intake and Se deficiency, along with Se speciation analysis will shed further light on the whether there is a potential relationship between Se, PTB risk and gestational duration.

## Data Availability

Data are available upon reasonable request. Deidentified participant data, the statistical code, and technical processes are available from the corresponding author on reasonable request.
