# Supplementary material for: Association of maternal prenatal selenium concentration and preterm birth: a multicountry meta-analysis
Source: BMJ Glob Health. 2021 Sep 9;6(9):e005856. doi: 10.1136/bmjgh-2021-005856 (PMC8438754; doi:10.1136/bmjgh-2021-005856)
Supplement: Supplementary data [file bmjgh-2021-005856supp001.pdf]

BMJ Publishing Group Limited (BMJ) disclaims all liability and responsibility arising from any reliance placed on this supplemental material which has been supplied by the author(s)

SUPPLEMENTARY MATERIAL

Table of Contents

Supplementary Text 1: Summary description of the parent study, site setting and sample collection.............2

Supplementary Text 2: Protocol for Selenium analysis in plasma and serum samples................................5

Supplementary Text 3: Regression analysis weighted by inverse of sampling probabilities..........................7

Supplementary Tables 8

Supplementary Table 1. Study characteristics of participant cohorts 8

Supplementary Table 2. Covariates (major phenotypes) requested from sites...9

Supplementary Table 3. Summary statistics of maternal Se concentration in different study sites 10

Supplementary Table 4. Demographic characteristics of Malawi study subjects 11

Supplementary Table 5. Summary statistics of Se concentration in Malawi study subjects 12

Supplementary Table 6. Demographic characteristics of UK (Liverpool) study subjects 13

Supplementary Table 7. Summary statistics of Se concentration in UK (Liverpool) study subjects 14

Supplementary Figures 15

Supplementary Figure 1: Distribution of gestational duration of singleton live births with spontaneous on set of labor from all sites 15

Supplementary Figure 2. Gestational duration in term and preterm deliveries by participating sites............16

Supplementary Figure 3. Correlation of gestational duration and preterm birth with other covariates 17

Supplementary Figure 4. Distribution of maternal Se concentration of samples from all sites 18

Supplementary Figure 5. Maternal Se concentration in term and preterm deliveries by participating sites 19

Supplementary Figure 6. Maternal Se concentration measured at different batches (colored by site) 20

Supplementary Figure 7. Correlation of maternal Se concentration with other covariates 21

Supplementary Figure 8. Gestational age (weeks) at sample collection by sites......................................22

Supplementary Figure 9. Meta-analysis of the association of maternal Se concentration with gestational duration with adjustment of case/control sampling with IPW analysis………...23

Supplementary Figure 10. Meta-analysis of the association of Selenium concentration (unit: 15 ng/ml) with PTB (A) and gestational duration (B) among the 4 Malawi sites 24

Supplementary References 25

Monangi N, *et al. BMJ Global Health* 2022; 6:e005856. doi: 10.1136/bmjgh-2021-005856

Supplementary Text

Supplementary Text 1: Summary description of the parent study, site setting and sample collection

Bangladesh (AMANHI) is a biobank in a population-based cohort of 3,000 pregnant women enrolled before 19 weeks of gestation and followed up-to 42 days post-partum. The overarching goal is to facilitate discoveries of biomarkers of adverse pregnancy outcomes (maternal, fetal and neonatal health outcomes) as new and more feasible methods become available. An additional goal is to identify biological mechanisms underlying the causes of the adverse outcomes including preeclampsia, spontaneous preterm birth (sPTB), stillbirth and intrauterine growth restrictions (IUGR) to create a platform to generate new approaches to treatment and prevention(1). All pregnant women were identified through pregnancy surveillance conducted by making home visits every 2 months by trained community health workers (CHWs). Pregnancies were confirmed via strip–based pregnancy tests and dated through ultrasound scans carried out by trained ultrasonologists before 19 weeks of gestation. The biobank contains maternal blood and urine specimens collected two times during pregnancy (8-19 weeks and 24-28 weeks or 32-36 weeks of gestation) and once during postpartum period (Day 42 postpartum) as well as delivery samples. Trained phlebotomists obtained maternal and umbilical cord blood samples and generated aliquots of serum, plasma, and buffy coats for storage. We also have collected and stored maternal urine, placental samples, umbilical cord blood, tissue and membrane. When cord blood collection was not possible, saliva was collected from newborns. In addition, we collected infant blood at 12 months of age and paternal saliva samples. All samples were processed and stored in -80°C freezers. CHWs collected detailed phenotypic and epidemiological data from the pregnant women four times during pregnancy (at 8-19 weeks, 24-28 weeks, 32-36 weeks, and 38-40 weeks of gestation), at delivery and twice during postpartum period (<7 days and at 42 days). The study started in July, 2014 and ended in April, 2018.

Bangladesh (GAPPS) is a population based prospective cohort study of pregnant women with an objective to establish infrastructure for researchers to conduct preterm birth and pregnancy related research. The study enrolled 4220 pregnant women from Matlab district over three years (2015-2017). The research site enrolled women early in pregnancy and collected information and biological specimens during their pregnancy and delivery. Gestational age was estimated by standardized ultrasound method. All biological samples were stored immediately in the local biorepository using the standardized protocol developed by GAPPS domestic biorepository. The study was approved by the research ethics committee of the Matlab Health Research Center at the International Center for Diarrheal Disease Research, Bangladesh.

Bangladesh (MDIG) study is a randomized intervention trial of vitamin D supplementation during pregnancy and lactation (2). Participants were generally healthy pregnant women between 17 and 24 weeks of gestation that were enrolled between March 2014 and September 2015 at the Maternal and Child Health Training Institute in Dhaka, Bangladesh. Gestational age was estimated by ultrasound or LMP or both. The study was approved by research ethics committees at the Hospital for Sick Children at Toronto and the International Center for Diarrheal Disease Research, Bangladesh (icddr,b).

Brazil, Kenya, Pakistan, South Africa, Thailand and UK (Interbio) is a multicenter, population-based research initiative coordinated by the University of Oxford to assess human growth, neurodevelopment and associated behaviors from early pregnancy to 2 years of age (3). The Interbio study was conducted between February 2012 and June 2018 at sites: Pelotas (Brazil), Nairobi (Kenya), Karachi (Pakistan), Soweto (South Africa), Mae Sot (Thailand) and Oxford (UK). Gestational age was determined by standardized ultrasound method. The studies were approved by regional ethics or institutional research boards and by the Institutional review board at the University of Oxford.

Malawi (iLiNS-DYAD) is a randomized, controlled, partially blinded, parallel-group intervention trial known as the International Lipid-based Nutrient Supplements DYAD trial which was designed to study the health impacts of lipid-based nutrient supplements during pregnancy and lactation (4). The study was conducted in 2 hospitals and 2 health centers in a rural area in Mangochi district between 2011-2012. Participants of the Malawi cohort were enrolled from four health facilities that covered mostly one continuous area near Lake Malawi. Lungwena, Malindi, and Mangochi subsites are along the banks of Lake Malawi and close to Namizimu forest reserve. Gestational age was determined using the ultrasound method. The ethical clearance for the study was granted by the University of

Malawi College of Medicine Research and Ethics Committee (COMREC) and the ethics committee at Tampere University Hospital District, Finland.

Pakistan (AMANHI) is a population-based biorepository with the aim of collecting cause-specific biosamples on maternal and neonatal mortality, and stillbirths from a well-characterized cohort of pregnant women(1). The biobank enrolled 2,500 pregnant women from 2014 to 2018 with the last pregnancy outcome occurring in January 2019, after an ultrasound in early pregnancy (<20 weeks) to confirm the gestational age. Women were also visited thrice at 24-28, 32-36 and 38-42 weeks gestation during pregnancy, and 0-6 days and 42-59 days after birth to measure blood pressure, test urine for proteinuria, and ascertain reported morbidity since the previous visit. The outcome of each identified pregnancy, whether abortion, stillbirth or live birth, was carefully documented and verbal autopsies were conducted with appropriate respondents in case of maternal deaths, stillbirths or neonatal deaths. Subsequently, all live born babies were assessed for neuromuscular, physical and feeding maturity as well as neonatal anthropometry (including baby's weight and foot length) within 72 hours of birth up until 4-5 years of age, using harmonized procedures. Blood and urine samples were collected from each woman at three time points: enrolment (<20 weeks), at either 24-28 weeks or 32-36 weeks gestation, and at 42 days postpartum. These samples were processed and stored at -80ºC in multiple aliquots. At delivery of a still- or live birth, maternal stool, umbilical cord blood and placental were collected, processed and stored within 30 minutes of birth. At the 42 days postpartum visit, maternal blood and urine, infant stool and paternal saliva samples were also collected for processing and storage. Infant saliva was collected if cord blood could not be obtained. Study protocols for enrolment, visits, ultrasound scans, sample collection, processing and storage were implemented by highly trained and motivated staff. Community members were found to be very cooperative and supportive of the study. The study was approved by the regional ethics review committee.

Tanzania (AMANHI) Tanzania (Pemba) is one of the AMANHI sites with bio-banked biological samples from a cohort of 4501 pregnant women and their children. The overall objective of all participating sites in the AMANHI study was similar, and all the SOP implemented for sample collection and processing were harmonized across sites(1). The study was conducted in Pemba island of the Zanzibar archipelago with an overall population of around 432000 and approximately 82,000 households. Two districts of the island were selected for the study where pregnancy surveillance was conducted every 2 months to identify pregnant women. Consent was obtained for confirmation of pregnancy by urine strip tests, thereafter gestational age was confirmed with routine ultrasound methods. All women between 8-19 weeks of gestation were consented and then enrolled in the study. Blood and urine samples were collected from the enrolled mothers at the time of enrollment and in either during 24 – 28 weeks or 32 – 36 weeks of gestation and 42 days postpartum. At delivery in addition to cord blood, tissue samples from placenta, membrane cord were collected within 30-60 minutes of delivery. All samples were processed as per harmonized SOP and stored in the biobank at -80^0 C. Saliva samples from the father and fecal samples from the mother and infant were also collected after delivery. For collecting the epidemiological data study team visited all the mothers during pregnancy and after delivery. Additional information on delivery was obtained from the hospital delivery records filled in by the physician in-charge. A in house designed AMANHI biobank LMIS software was used by all AMANHI sites for recording the collection, processing and storage information of the biospecimens. Stringent quality control checks were implemented for data consistency and sample quality during the study. The study started in June 2014 and the last postnatal follow-up sample from the mother was collected in December 2018.

UK (Liverpool) was started in April 2012 and ended in December 2017. The study entitled "The development of novel biomarkers for prediction of preterm labor in a high-risk population". This study enrolled a total of 541 pregnant women with singleton pregnancies at the Liverpool Women's Hospital, UK. Gestational age was determined using the ultrasound method. The study was approved by the Institutional Review board at the University of Liverpool.

USA, CA(CPPOP) is a nested case-control sampling study drawn from a population-based cohort of 757,853 singleton live births in the state of California (5). Women with nonfasted serum samples banked by the California biobank program from July 2009 through December 2010 were enrolled in the study. Gestational age was determined by ultrasound or LMP or both. Methods and protocols for the study were approved by the Committee for the Protection of Human Subjects within the Health and Human Services Agency of the State of California, the

Institutional Review Board of Stanford University and the Institutional Review Board of the University of California San Francisco.

USA, NC (NEST) is a perinatal cohort study of more than 2000 pregnant and their offspring mounted to investigate the role of environmental exposures and nutrition in utero on the shifts in the epigenome of newborns (6), from which we nested a case control study. The study participants were a birth cohort from women who received prenatal care in the Duke/Durham region health care system in Durham, NC between 2005 and 2009. Ultrasound is used to determine the gestational age. The study was approved by the Institutional Review Board of Duke University, North Carolina.

Vietnam (PBB) is an observational study in Ho Chi Minh city to evaluate biomarkers for spontaneous preterm birth in partnership with Sera Prognostics, OUCRU and the Gates Foundation. This is a prospective hospital-based convenience sampling of 4800 women between September 2016 through August 2018 who have antenatal care and plan to deliver at Tu Du hospital and were between 19^{+0} to 22^{+6} days of gestation when samples were taken. Gestational age was determined using ultrasound. The study was approved by the Institutional review board at the Tu Du Hospital and the University of Oxford (OXTREC 28-16).

Zambia (GAPPS) is a population based prospective cohort study of pregnant women with an objective to establish infrastructure for researchers to conduct preterm birth and pregnancy related research. The study enrolled 2000 pregnant women from Lusaka district over three years (2015-2017). The research site enrolled women early in pregnancy and collect information and biological specimens during their pregnancy and delivery. Gestational age was estimated by ultrasound at gestational weeks 16-22. All biological samples were stored immediately in the local biorepository using the standardized protocol developed by GAPPS domestic biorepository. The study was approved by the regional ethics review board.

Supplementary Text 2: Protocol for Selenium analysis in plasma and serum samples

Sample Preparation

Before analysis the serum and plasma samples were thawed on ice for 30 minutes followed by sonication in an ultrasonic bath (Fisher Scientific, CPX1800) for 5 minutes in order to mix the samples. During these processes, the sample vials remained sealed in order to prevent dilution of the sample caused by the premature opening of the sample vials before they reach room temperature. An acid digestion was performed on the samples with the following protocol: 50 µL of plasma or serum from each sample as well as the quality control serum was transferred to 15 mL metal free vials (VWR) using clear pipette tips and a calibrated electronic micropipette (Eppendorf). Both the samples and the quality control serum were mixed briefly using a mini vortex mixer (VWR) immediately prior to transferring the aliquot in order to promote better sampling of a nonhomogeneous sample matrix. The quality control serum used was obtained from UTAK Laboratories, Inc. and included normal range trace elements which was reconstituted according to manufacturer instructions. In the event that 50 uL of sample could not be obtained, 40 uL or as low as 30 uL aliquots of sample and quality control serum were used according to sample availability.

50 µL of the internal standard mixture containing 500 ppb of Sc, In, Y and Te in 0.5 M nitric acid (High Purity Standards) was then added with a repetition pipette (Eppendorf) to the 15 mL metal free vials, followed by 200 µL of concentrated trace metal grade nitric acid (Sigma-Aldrich; Fisher Scientific). The samples were placed in a dry bath with no more than 3 cm of the tubes immersed in the heating block holes in order to allow reflux of the sample. For this, aluminum foil was inserted into the block holes.

The samples were heated at 85 ºC for one hour, then at 95 ºC for 1.5 hours. In order to complete the digestion, 100 µl of trace metal grade hydrogen peroxide (Sigma-Aldrich) was added with a repetition pipette after the samples cooled for 5 minutes outside the heating block. Then the samples were returned to the heating block another 30 minutes at 95 ºC. Once the digestion was completed, the final volume was brought up to 2.5 ml with doubly deionized water and mixed using a vortex mixer.

**Final Concentration of Standard (ppb)**	**0**	**0.5**	**1**	**2**	**5**	**10**	**25**
Volume of 100 ppb Working Standard (µL)	0	25	50	100	250	500	1250
Volume of 500 ppb Internal Standard (µL)	100	100	100	100	100	100	100
Volume of 0.5M HNO3 (µL)	4900	4875	4850	4800	4650	4400	3650

Protocol table 1. Calibration standard preparation

Calibration Preparation

The external calibration method with internal standard in-samples was used. For this the following points were used: 0 ppb, 0.5 ppb, 1 ppb, 2 ppb, 5 ppb, 10 ppb and 25 ppb. The individual volumes used are shown in Protocol supplementary table 1. The standards were prepared in 0.5 M trace metal grade nitric acid. A stock solution containing a mixture of elements of interest at 10 ppm (SPEX CertiPrep^{TM}) was used to prepare a daily working standard of 100 ppb. For this a 100x dilution was made by adding 50 µL of the stock standard to a metal free vial and adding 0.5 M trace metal grade nitric acid to 5 mL. The final volume for the calibration standards was 5 mL. 100 µL of internal standard mixture was added to each calibration standard.

Instrumentation

The instrument used for selenium analysis was an Agilent 7700 ICP-MS. The use of a collision/reaction cell with hydrogen is essential for the proper detection power and long-term signal stability of Se in the samples required for the analysis of long batches. The ^{77}Se and ^{78}Se isotopes along with the Internal Stand Isotope ^{125}Te were monitored with one second of integration time and three replicates in hydrogen mode. A set of typical calibration results is shown in **Protocol figure 1**.

In the sequence, a set of heated 0.5 M nitric acid blanks with internal standard were added every 60 samples, a repetition of the 1 ppb calibration standard was also included every 60 samples and the last sample to be run was a repeat of the digested quality control serum. Two non-treated certified reference materials were used to validate the accuracy of the calibration curve with a 20x dilution, Trace Metals in Drinking Water (CRM TMDW-B, High Purity Standards) and River Sediment Solution (CRM-RS-A, High Purity Standards). This is important in order to identify the source of error in the case that the digested serum CRM values were not obtained. If the non-digested samples also show error, then the calibration or instrument performance was assumed to be the problem; while if the non-digested CRMs showed good results, then the problem was assumed to be with the digestion of the samples.

Supplementary Text 3: Regression analysis weighted by inverse of sampling probabilities.

Eight cohorts included in this study used case/control sampling (Supplementary Table 1). It is known that non-random sampling could introduce bias in the estimation of the effect size on the secondary quantitative outcomes (i.e. gestational duration). To examine whether this problem can influence our analysis, we conducted regression analysis adjusted by inverse probability weights (IPW) and compared the result with the naïve analysis (without adjustment of case/control sampling). The IPW analysis corrects for selection bias by weighting the observations by the inverse of the sampling probabilities based on their case/control status. Specifically, for a case/control data set with case: control ratio (r), the relative sampling probability of controls (sampling probability of cases was set to 1) was calculated as $k/(1-k)/r$, where k is the disease prevalence of the target population or the frequency of cases in the parental random cohort. The inverse of the sampling probabilities was then used as weights in the regression analysis to correct for the sampling bias of case/control data.

SUPPLEMENTARY TABLES

Supplementary Table 1. Study characteristics of participant cohorts

Site	Location	Study design, sample collection	Year	Data sharing format with CCHMC	GA estimation method		Type of Sample
					Ultrasound	LMP	
Bangladesh (AMANHI)	Sylhet	Population based, random	2012-2016	Case:Control (1:1)	X		Plasma
Bangladesh (GAPPS)	Matlab	Population based, random	2015-2017	Case:Control (1:2)	X		Serum
Bangladesh (MDIG)	Dhaka	Hospital based, intervention trial	2014-2015	Case:Control (1:2)	X	X	Serum
Brazil (INTERBIO)	Pelotas	Hospital based, random	2009-2014	Random	X		Plasma
Kenya (INTERBIO)	Nairobi	Hospital based, random	2009-2014	Random	X		Plasma
Malawi (iLiNS-DYAD)	Mangochi	Hospital based, intervention trial	2011-2015	Random	X		Plasma
Pakistan (AMANHI)	Karachi	Population based, random	2012-2016	Case:Control (1:2)	X		Serum
Pakistan (INTERBIO)	Karachi	Hospital based, random	2009-2014	Random	X		Plasma
South Africa (INTERBIO)	Johannesburg	Hospital based, random	2009-2014	Random	X		Plasma
Tanzania (AMANHI)	Pemba	Population based, random	2009-2016	Case:Control (1:2)	X		Plasma
Thailand (INTERBIO)	Mae Sot	Hospital based, random	2009-2014	Random	X		Plasma
UK (INTERBIO)	Oxford	Hospital based, random	2009-2014	Random	X		Plasma
UK (LIVERPOOL)	Liverpool	Hospital based, targeted recruitment	2016-2017	Random	X		Plasma
USA, CA (CPPOP)	San Francisco	Hospital based, nested case-control	2009-2010	Case:Control (1:1)	X	X	Serum
USA, NC (NEST)	Durham	Hospital based, random	2005-2009	Case:Control (1:2)	X		Plasma
Vietnam (PBB)	Ho Chi Minh City	Hospital based, random	2016-2018	Case:Control (1:2)	X	X	Serum
Zambia (GAPPS)	Lusaka	Hospital based, random	2015-2017	Random	X		Serum

Monangi N, *et al. BMJ Global Health* 2022; 6:e005856. doi: 10.1136/bmjgh-2021-005856

Supplementary Table 2. Covariates (major phenotypes) requested from sites

Variable	Required	Desired
**Baseline characteristics**		
Gestational Age (at time of sample collection)	x	
Maternal Age		x
Maternal race		x
Maternal ethnicity		x
**Birth history**		
Gravidity (# of pregnancies)		x
Parity (# of births)		x
# prior PTB		x
# prior stillbirth		x
**Pre-pregnancy BMI**		
Height/weight at visit	x	
Pre-pregnancy weight (if available)	x	
**Exposures**		
Smoking during pregnancy		x
Alcohol during pregnancy		x
Substance use during pregnancy		x
**Delivery outcomes**		
Delivery date		x
Gender	x	
Gestational age at delivery	x	
Birth weight	x	
Spontaeous versus indicated delivery (if available)	x	
Infant/fetus vital status (live birth)	x	
**Conditions**		
Chorioamnionitis		x
Hypertensive disorder		x
Preeclampsia		x
Gestational diabetes		x
Other specified conditions		x

Monangi N, *et al. BMJ Global Health* 2022; 6:e005856. doi: 10.1136/bmjgh-2021-005856

Supplementary Table 3. Summary statistics of maternal Se concentration in different study sites

	Se concentration (ng/ml)							
**site**	**Min.**	**1st Qu.**	**Median**	**Mean**	**3rd Qu.**	**Max.**	**sd**	**N**
Bangladesh (AMANHI)	48.1	91.9	104.8	108	120.5	219.5	23.4	506
Bangladesh (GAPPS)	48.1	67.6	74.5	74.8	80.3	117.2	10.6	258
Bangladesh (MDIG)	48.5	67.8	74.4	75.3	82.3	108.8	11.4	206
Brazil (INTERBIO)	33.2	50.3	57.6	58.7	64.9	146	12.6	389
Kenya (INTERBIO)	68.1	89.9	97.5	97.9	105.8	142.1	11.9	553
Malawi (iLiNS-DYAD)	26.1	61.8	78.2	83	97.5	228.7	29.5	1210
Pakistan (AMANHI)	71	93	102.4	102.8	111.6	157.6	13.8	348
Pakistan (INTERBIO)	53.7	82	88.7	89.1	95.3	187.6	12.2	516
South Africa (INTERBIO)	28.6	64.3	70.9	70.6	77.5	109.6	10.3	352
Tanzania (AMANHI)	77.7	114.1	129.8	131.4	144.9	223.1	23.8	351
Thailand (INTERBIO)	61.4	93.3	103.7	105.4	115.8	172.2	17.3	514
UK (INTERBIO)	58	82.5	90.6	90.9	97.8	157.2	12.8	648
UK (Liverpool)	40	69.5	79.3	80	89	177.7	15.8	525
USA, California (CPPOP)	74.7	115.9	125.2	125.3	134	215.5	15.3	966
USA, North Carolina (NEST)	61.7	112.3	125.2	125.6	138.7	204.4	20.6	657
Vietnam (PBB)	59.5	90.2	99.3	102.4	111	182.5	18.3	970
Zambia (GAPPS)	29.8	49.1	55.6	55.9	61.9	105.3	9.8	973
Total	26.1	72.6	92.3	93.8	112.8	228.7	28.5	9942

Monangi N, *et al. BMJ Global Health* 2022; 6:e005856. doi: 10.1136/bmjgh-2021-005856

BMJ Publishing Group Limited (BMJ) disclaims all liability and responsibility arising from any reliance placed on this supplemental material which has been supplied by the author(s)

Supplementary Table 4. Demographic characteristics of Malawi study subjects

Site	Sample Size	Term	Preterm	Male	Female	Gday at delivery	Gday at sampling	Maternal Age (year)	Maternal Height (cm)	Birth Weight (g)
Lungwena	473	448 (95%)	25 (5%)	242 (51%)	231 (49%)	276.3 (13.2)	117.3 (13.9)	25.5 (6.4)	155.7 (5.6)	2948.8 (427.6)
Malindi	240	229 (95%)	11 (5%)	110 (46%)	130 (54%)	280.7 (14)	117.5 (15.6)	24.5 (5.9)	156.9 (5.9)	3129.3 (454.5)
Namwera	187	161 (86%)	26 (14%)	80 (43%)	107 (57%)	268.8 (15.8)	115.1 (16.2)	24.5 (6)	155.7 (5.9)	2918.3 (408.3)
Mangochi	312	288 (92%)	24 (8%)	155 (50%)	157 (50%)	276.2 (13.6)	120.3 (14.7)	25.5 (6)	156.5 (5.5)	2926.3 (474.3)
Total	1212	1126 (92.9%)	86 (7.1%)	587 (48.4%)	625 (51.6%)	276 (14.3)	117.7 (14.9)	25.2 (6.2)	156.1 (5.7)	2976.6 (449.5)

* Categorical variables are shown as count (percentage) and continuous variables are shown as mean (sd).

* Gday: gestational days. Term ≥ 259 days and Preterm: gday < 259 days.

Supplementary Table 5. Summary statistics of Se concentration in Malawi study subjects

	Se concentration (ng/ml)							
**Site**	**Min.**	**1st Qu.**	**Median**	**Mean**	**3rd Qu.**	**Max.**	**sd**	**N**
**Lungwena**	32.7	63.3	82.1	88.1	105.3	228.7	32.4	472
**Malindi**	46.6	76	91.3	94.8	109.3	174.4	24.6	239
**Namwera**	26.1	46.1	56.2	56.8	67.6	94.7	14.4	187
**Mangochi**	31.5	65.2	78	82	93.3	195.3	25.4	312
**Total**	26.1	61.8	78.2	83	97.5	228.7	29.5	1210

Supplementary Table 6. Demographic characteristics of UK (Liverpool) study subjects

Group	Sample Size	Term	Preterm	Male	Female	GA at delivery	GA at sampling	Maternal Age	Maternal Height	Birth Weight
Low-risk	253	249 (98%)	4 (2%)	125 (49%)	128 (51%)	276.4 (11.5)	141.2 (10.1)	30.9 (4.6)	165.5 (6.1)	3472.6 (495.2)
High-risk	272	175 (64%)	97 (36%)	146 (54%)	126 (46%)	258.3 (25)	140.5 (8.8)	30.3 (5.1)	164.2 (6.5)	2830.8 (778)
Total	525	424 (80.8%)	101 (19.2%)	271 (51.6%)	254 (48.4%)	267 (21.7)	140.8 (9.5)	30.6 (4.9)	164.8 (6.3)	3141.2 (730.3)

* Categorical variables are shown as count (percentage) and continuous variables are shown as mean (sd).

* Gday: gestational days. Term ≥ 259 days and Preterm: gday < 259 days.

Supplementary Table 7. Summary statistics of Se concentration in UK (Liverpool) study subjects

	Se concentration (ng/ml)							
**Site**	**Min.**	**1st Qu.**	**Median**	**Mean**	**3rd Qu.**	**Max.**	**sd**	**N**
Low-risk	51.0	71.3	82.2	82.7	92.3	133.1	15.6	253
High-risk	40.0	67.7	76.6	77.6	85.6	177.7	15.6	272
Total	40.0	69.5	79.3	80.0	89.0	177.7	15.8	525

SUPPLEMENTARY FIGURES

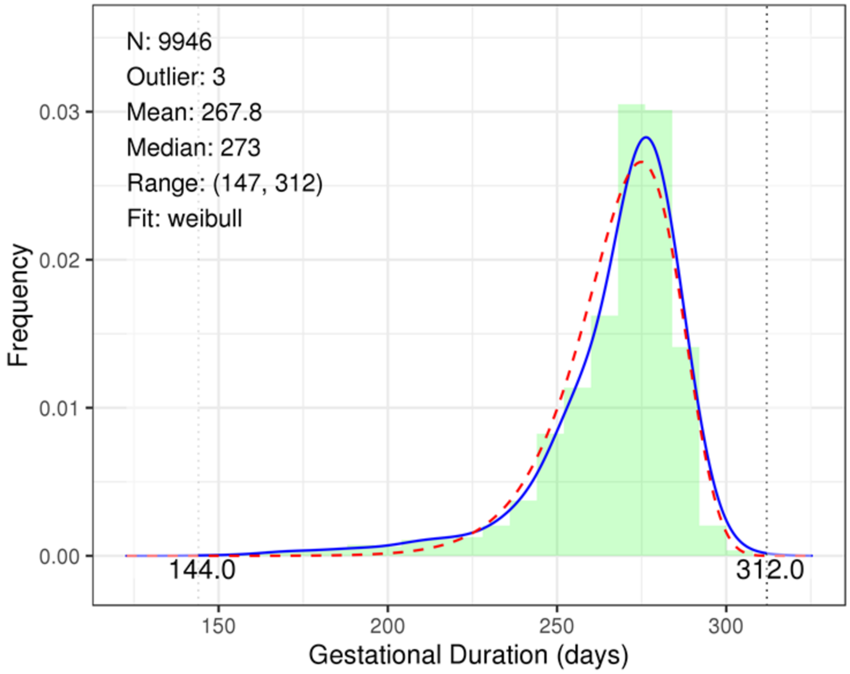

Supplementary Figure 1: Distribution of gestational duration of singleton live births with spontaneous on set of labor from all sites

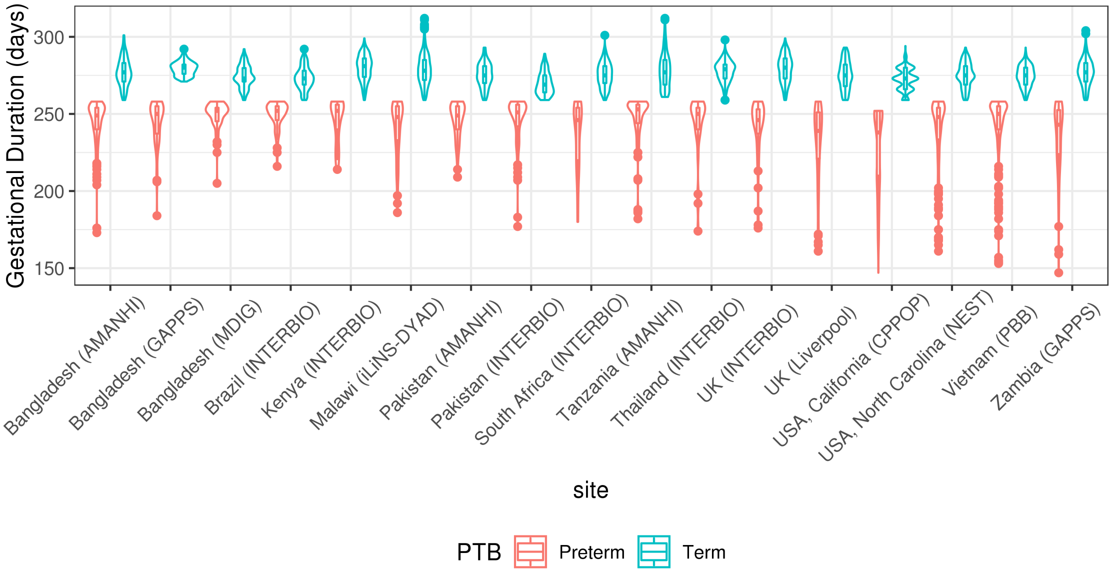

Supplementary Figure 2. Gestational duration in term and preterm deliveries by participating sites

Violin plot illustrating the distributions of gestational days in term (gday ≥ 259 days) and preterm (gday < 259 days) deliveries from each site.

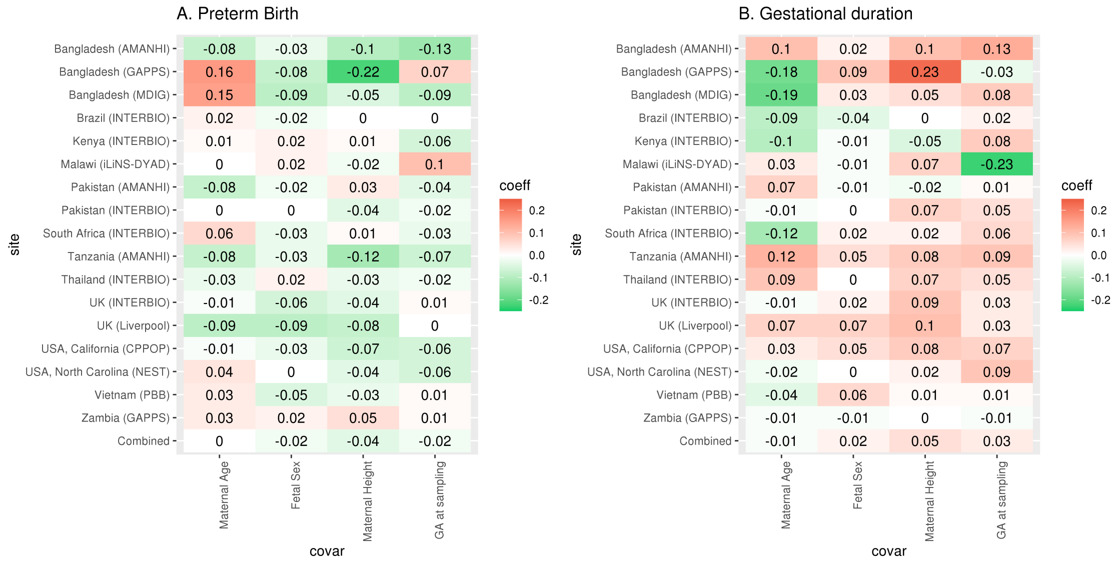

Supplementary Figure 3. Correlation of gestational duration and preterm birth with other covariates

Heat maps illustrating the correlation of preterm birth (A) and gestational duration (B) with pregnancy covariates. Red shading indicates a positive correlation and green a negative correlation, with intensity reflecting the magnitude of the correlation.

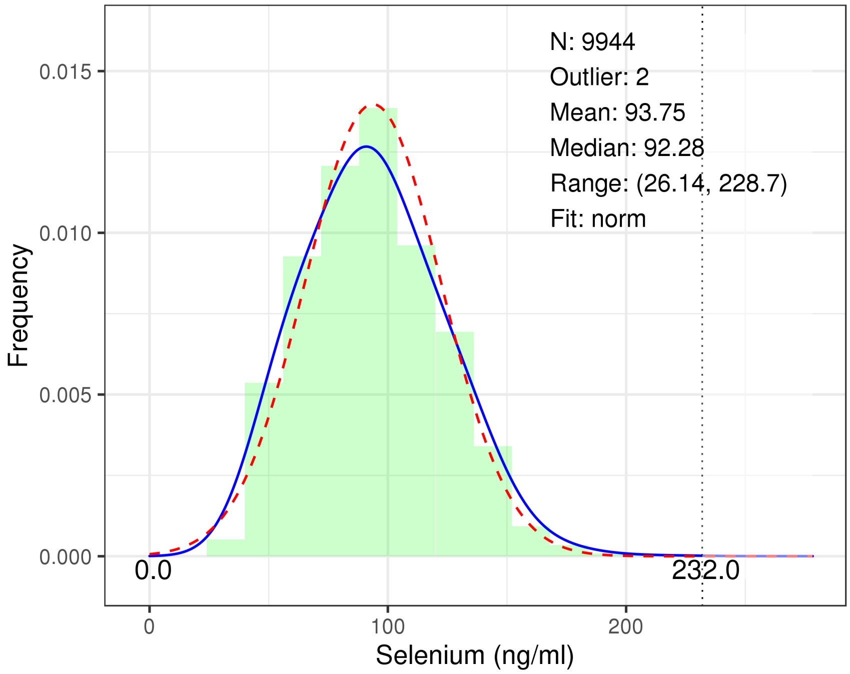

Supplementary Figure 4. Distribution of maternal Se concentration of samples from all sites

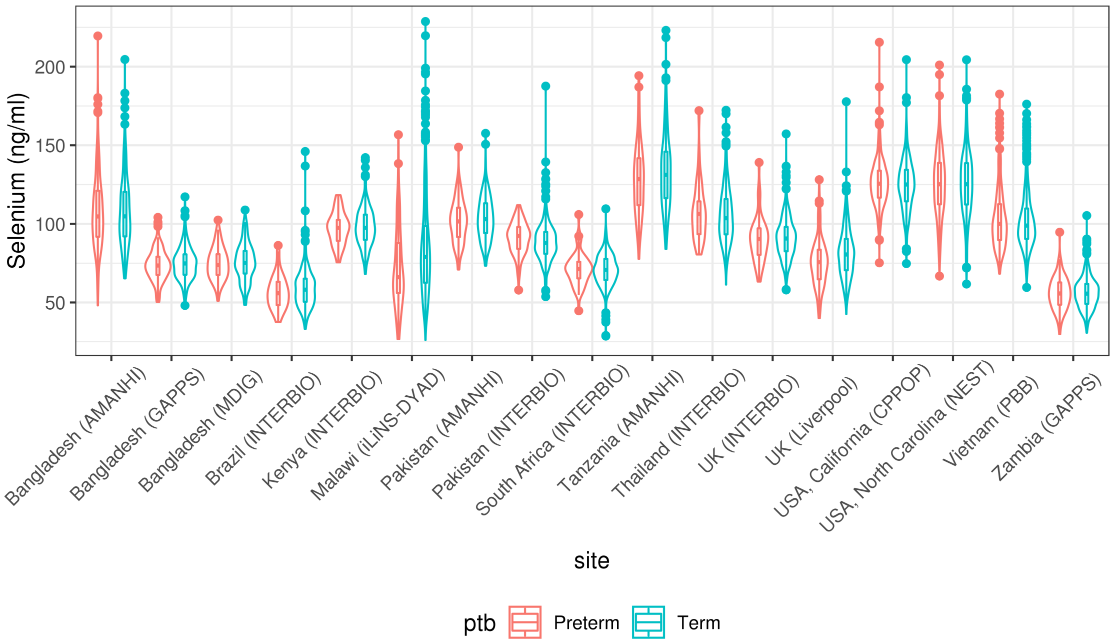

Supplementary Figure 5. Maternal Se concentration in term and preterm deliveries by participating sites

Violin plot illustrating the distributions of Se concentration in term (gday ≥ 259 days) and preterm (gday < 259 days) deliveries from each site.

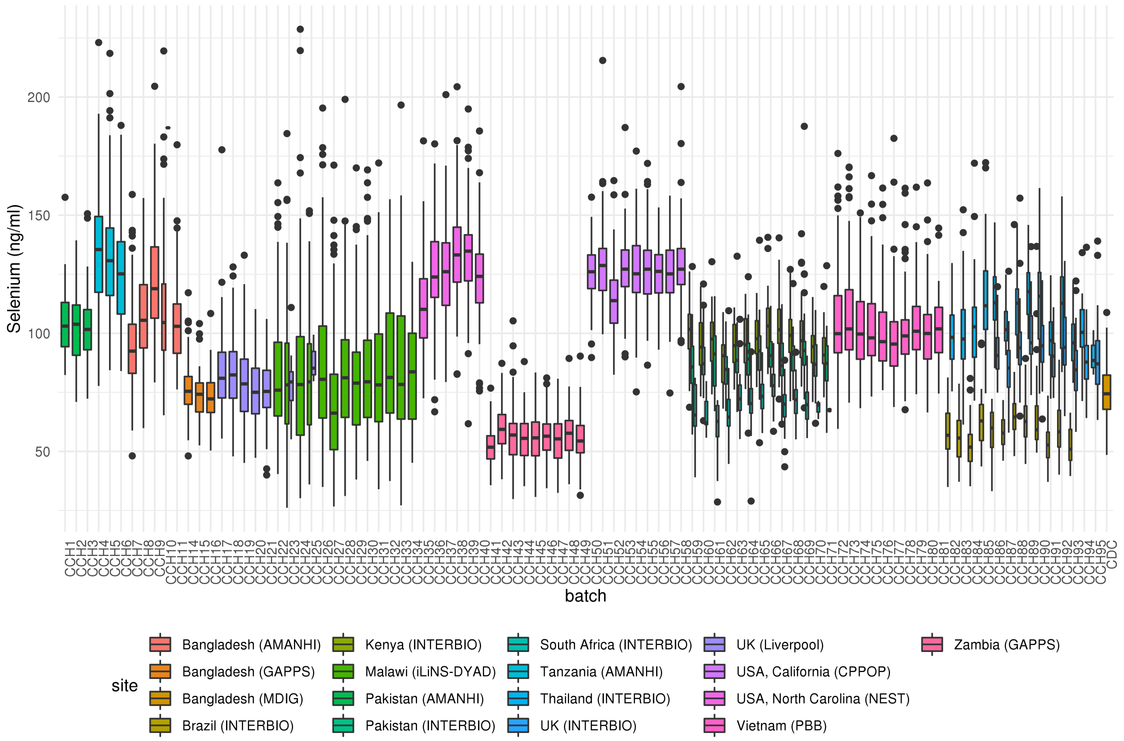

Supplementary Figure 6. Maternal Se concentration measured at different batches (colored by site)

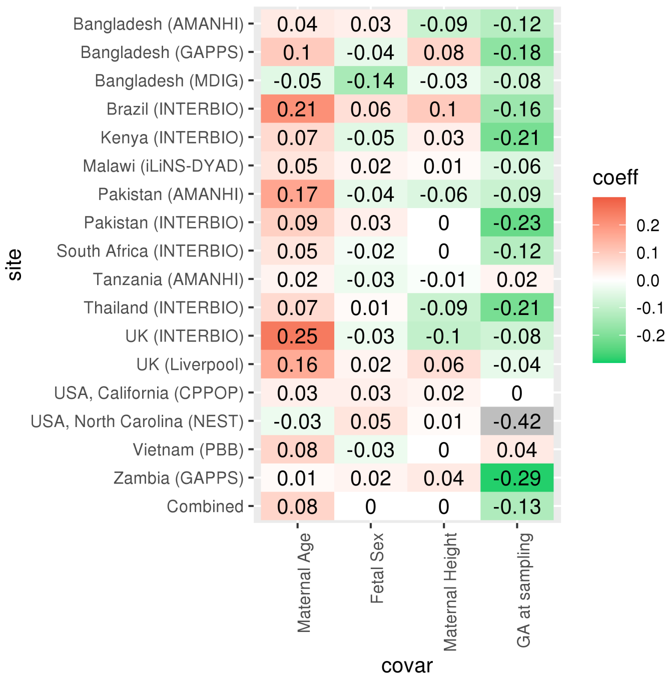

Supplementary Figure 7. Correlation of maternal Se concentration with other covariates

Heat maps illustrating the correlation of maternal Se concentration with other covariates. Red shading indicates a positive correlation and green a negative correlation, with intensity reflecting the magnitude of the correlation.

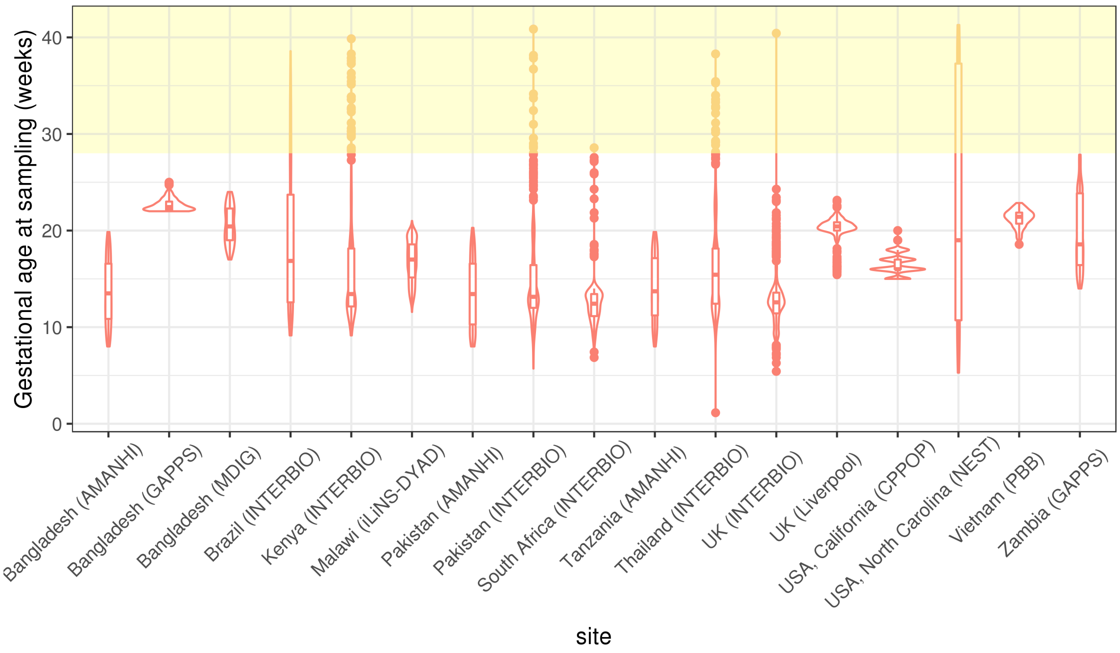

Supplementary Figure 8. Gestational age (weeks) at sample collection by sites

Violin plot illustrating the distribution of gestational age (weeks) at sample collection by sites. Yellow shaded region represents the samples collect after 2nd trimester (≥ 28 wks), which were excluded from the final association analysis.

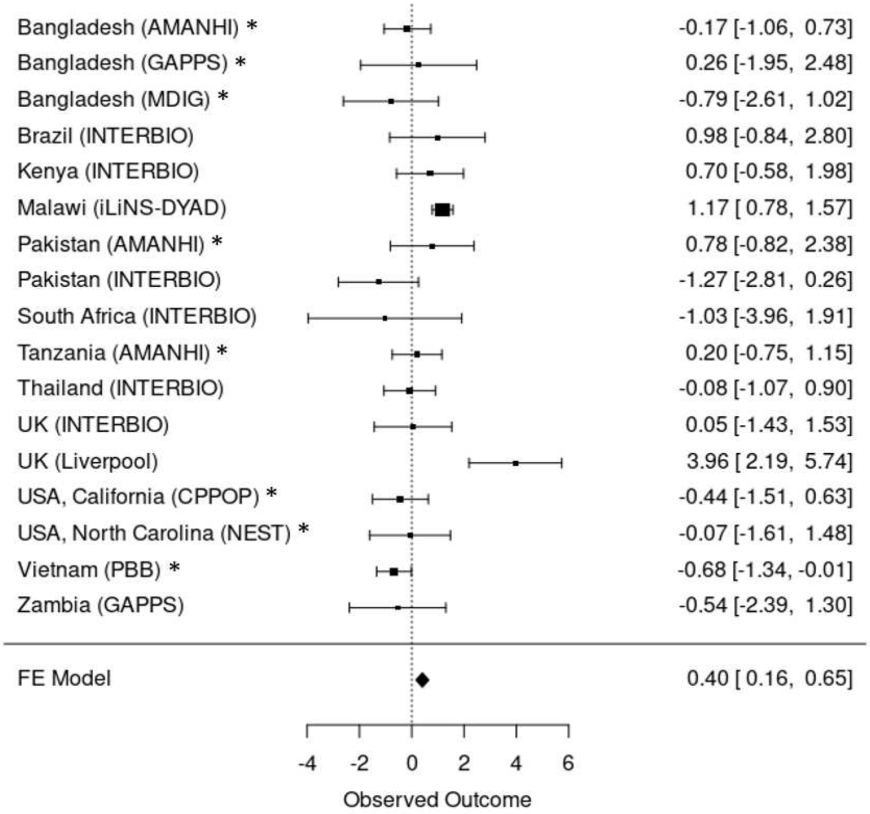

Supplementary Figure 9. Meta-analysis of the association of maternal Se concentration with gestational duration with adjustment of case/control sampling with IPW analysis.

The sites labeled with * were case/control data sets and the sampling bias were corrected using regression analysis weighted by inverse of sampling probabilities (Supplementary Text 3).

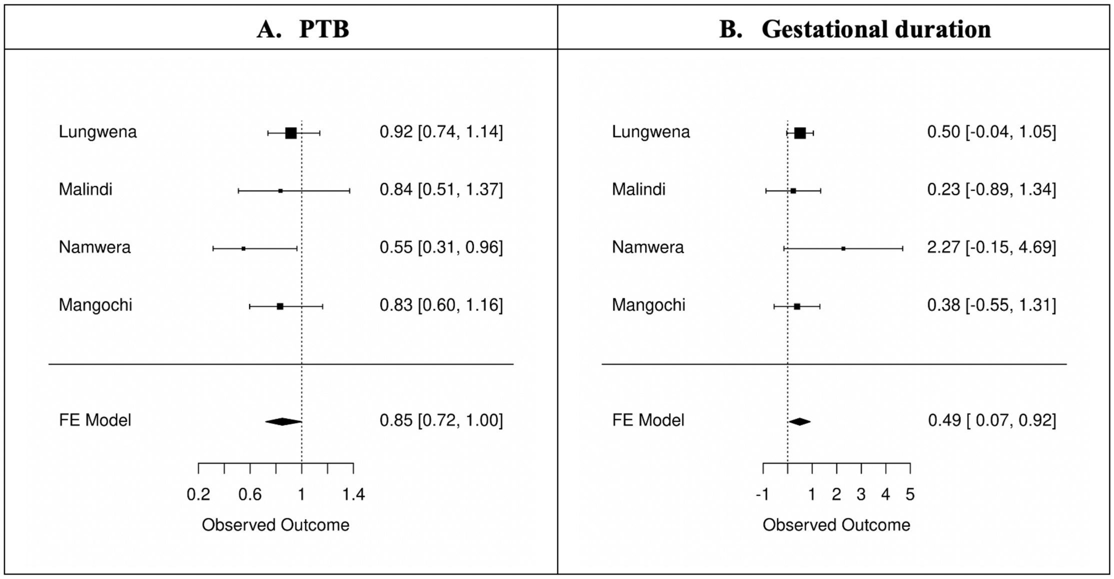

Supplementary Figure 10. Meta-analysis of the association of Selenium concentration (unit: 15 ng/ml) with PTB (A) and gestational duration (B) among the 4 Malawi sites

(A) The estimated effect on PTB is shown as odds ratio per 15 ng/ml increase in Se concentration. (B) The estimated effect on gestational duration is shown as change in gestational days per 15 ng/ml increase in Se concentration.

SUPPLEMENTARY REFERENCES

1. Alliance for M, Newborn Health Improvement mortality study g. Population-based rates, timing, and causes of maternal deaths, stillbirths, and neonatal deaths in south Asia and sub-Saharan Africa: a multi-country prospective cohort study. Lancet Glob Health. 2018;6(12):e1297-e308.
2. Roth DE, Morris SK, Zlotkin S, Gernand AD, Ahmed T, Shanta SS, et al. Vitamin D Supplementation in Pregnancy and Lactation and Infant Growth. N Engl J Med. 2018;379(6):535-46.
3. Kennedy SH, Victora CG, Craik R, Ash S, Barros FC, Barsosio HC, et al. Deep clinical and biological phenotyping of the preterm birth and small for gestational age syndromes: The INTERBIO-21 (st) Newborn Case-Control Study protocol. Gates Open Res. 2018;2:49.
4. Kamng'ona AW, Young R, Arnold CD, Patson N, Jorgensen JM, Kortekangas E, et al. Provision of Lipid-Based Nutrient Supplements to Mothers During Pregnancy and 6 Months Postpartum and to Their Infants from 6 to 18 Months Promotes Infant Gut Microbiota Diversity at 18 Months of Age but Not Microbiota Maturation in a Rural Malawian Setting: Secondary Outcomes of a Randomized Trial. J Nutr. 2020;150(4):918-28.
5. Jelliffe-Pawlowski LL, Rand L, Bedell B, Baer RJ, Oltman SP, Norton ME, et al. Prediction of preterm birth with and without preeclampsia using mid-pregnancy immune and growth-related molecular factors and maternal characteristics. J Perinatol. 2018;38(8):963-72.
6. Martin CL, Jima D, Sharp GC, McCullough LE, Park SS, Gowdy KM, et al. Maternal pre-pregnancy obesity, offspring cord blood DNA methylation, and offspring cardiometabolic health in early childhood: an epigenome-wide association study. Epigenetics. 2019;14(4):325-40.
